# TRAF3IP2, a novel therapeutic target in glioblastoma multiforme

**DOI:** 10.18632/oncotarget.25710

**Published:** 2018-07-03

**Authors:** Eckhard U. Alt, Zahra Barabadi, Andreas Pfnür, Joana E. Ochoa, Fatemeh Daneshimehr, Lea M. Lang, Dong Lin, Stephen E. Braun, Bysani Chandrasekar, Reza Izadpanah

**Affiliations:** ^1^ Applied Stem Cell Laboratory, Medicine/Heart and Vascular Institute, Tulane University Health Sciences Center, New Orleans, Louisiana, USA; ^2^ Department of Surgery, Tulane University Health Science Center, New Orleans, Louisiana, USA; ^3^ Division of Regenerative Medicine, Tulane National Primate Research Center, Covington, Louisiana, USA; ^4^ Department of Medicine, University of Missouri School of Medicine and Harry S. Truman Veterans Memorial Hospital, Columbia, Missouri, USA

**Keywords:** TRAF3IP2, glioblastoma multiforme, tumor microenvironment, cancer stem cells, inflammation

## Abstract

Glioblastoma multiforme (glioblastoma) remains one of the deadliest cancers. Pro-inflammatory and pro-tumorigenic mediators present in tumor microenvironment (TME) facilitate communication between tumor cells and adjacent non-malignant cells, resulting in glioblastoma growth. Since a majority of these mediators are products of NF-κB- and/or AP-1-responsive genes, and as TRAF3 Interacting Protein 2 (TRAF3IP2) is an upstream regulator of both transcription factors, we hypothesized that targeting TRAF3IP2 blunts tumor growth by inhibiting NF-κB and pro-inflammatory/pro-tumorigenic mediators. Our *in vitro* data demonstrate that similar to primary glioblastoma tumor tissues, malignant glioblastoma cell lines (U87 and U118) express high levels of TRAF3IP2. Silencing TRAF3IP2 expression inhibits basal and inducible NF-κB activation, induction of pro-inflammatory mediators, clusters of genes involved in cell cycle progression and angiogenesis, and formation of spheroids. Additionally, silencing TRAF3IP2 significantly increases apoptosis. *In vivo* studies indicate TRAF3IP2-silenced U87 cells formed smaller tumors. Additionally, treating existing tumors formed by wild type U87 cells with lentiviral TRAF3IP2 shRNA markedly regresses their size. Analysis of residual tumors revealed reduced expression of pro-inflammatory/pro-tumorigenic/pro-angiogenic mediators and kinesins. In contrast, the expression of IL-10, an anti-inflammatory cytokine, was increased. Together, these novel data indicate that TRAF3IP2 is a master regulator of malignant signaling in glioblastoma, and its targeting modulates the TME and inhibits tumor growth by suppressing the expression of mediators involved in inflammation, angiogenesis, growth, and malignant transformation. Our data identify TRAF3IP2 as a potential therapeutic target in glioblastoma growth and dissemination.

## INTRODUCTION

Glioblastoma multiforme (GBM/glioblastoma) is the most aggressive and diffuse brain tumor of astrocytic lineage. Despite aggressive interventions, including surgical resection followed by radiation therapy with concurrent chemotherapy with temozolomide [1], the survival remains poor.

In addition to malignant cells, glioblastoma lesions contain non-malignant cells that include endothelial cells, inflammatory cells, cells with stem-like properties, and cells with neural, glial, or myeloid markers. These cells, together with soluble inflammatory mediators, form the tumor microenvironment (TME). Reports have demonstrated the presence of complex communication networks between tumor and non-tumor cells within the TME that contribute to tumor growth and metastasis [2]. In fact, the pro-inflammatory mediators secreted by tumor and non-tumor cells contribute to their communication via both autocrine and paracrine mechanisms, resulting in further tumor growth [3–5]. For example, non-tumor cell-derived TNF-α, IL-6, IL-8, and IL-1β stimulate tumor cell proliferation, invasion, and metastasis via paracrine signaling [6–10]. These inflammatory mediators also possess chemotactic properties and recruit various immune cells to the TME. Increased numbers of inflammatory stromal and senescent cells further exacerbate inflammation and tumor growth [2, 5, 11]. Importantly, many of these inflammatory mediators are NF-κB- and/or AP-1-responsive genes.

Persistent activation of NF-κB amplifies inflammation and promotes tumor growth [12]. In fact, its persistent activation in glioblastoma confers poor prognosis [13]. In addition, the crosstalk between AP-1, STAT3, p53, HIF-1α, PPAR-γ, and β-catenin contribute to tumor growth and malignancy by enhancing the expression of growth factors, inflammatory mediators, anti-apoptotic proteins, and cell cycle regulators [14]. Glioblastoma cells secrete high levels of IL-8, a potent chemoattractant that promotes immune cell infiltration and inflammation in the TME [15]. Increased expression and secretion of TNF-α by glial cells promote tumor growth by amplifying inflammation and neoangiogenesis [16–18]. IL-1β, IL-6 and RANK Ligand also amplify inflammation and promote tumor growth and dissemination [19]. By upregulating HIF-1α, IL-1β activates NF-κB, and induces pro-angiogenic VEGF expression [20].

TRAF3 Interacting Protein 2 (TRAF3IP2) is an upstream regulator of NF-κB activation [21, 22]. It plays a critical role in IL-17 signaling. Its forced expression, by itself, activates multiple pro-inflammatory signaling pathways, including activation of NF-κB. Importantly, its increased expression plays a causal role in various autoimmune and inflammatory diseases. However, its causal role in glioblastoma is not known.

## RESULTS

### TRAF3IP2 expression is increased in primary human glioblastoma tumors and malignant glioblastoma cell lines

**Figure 1:**
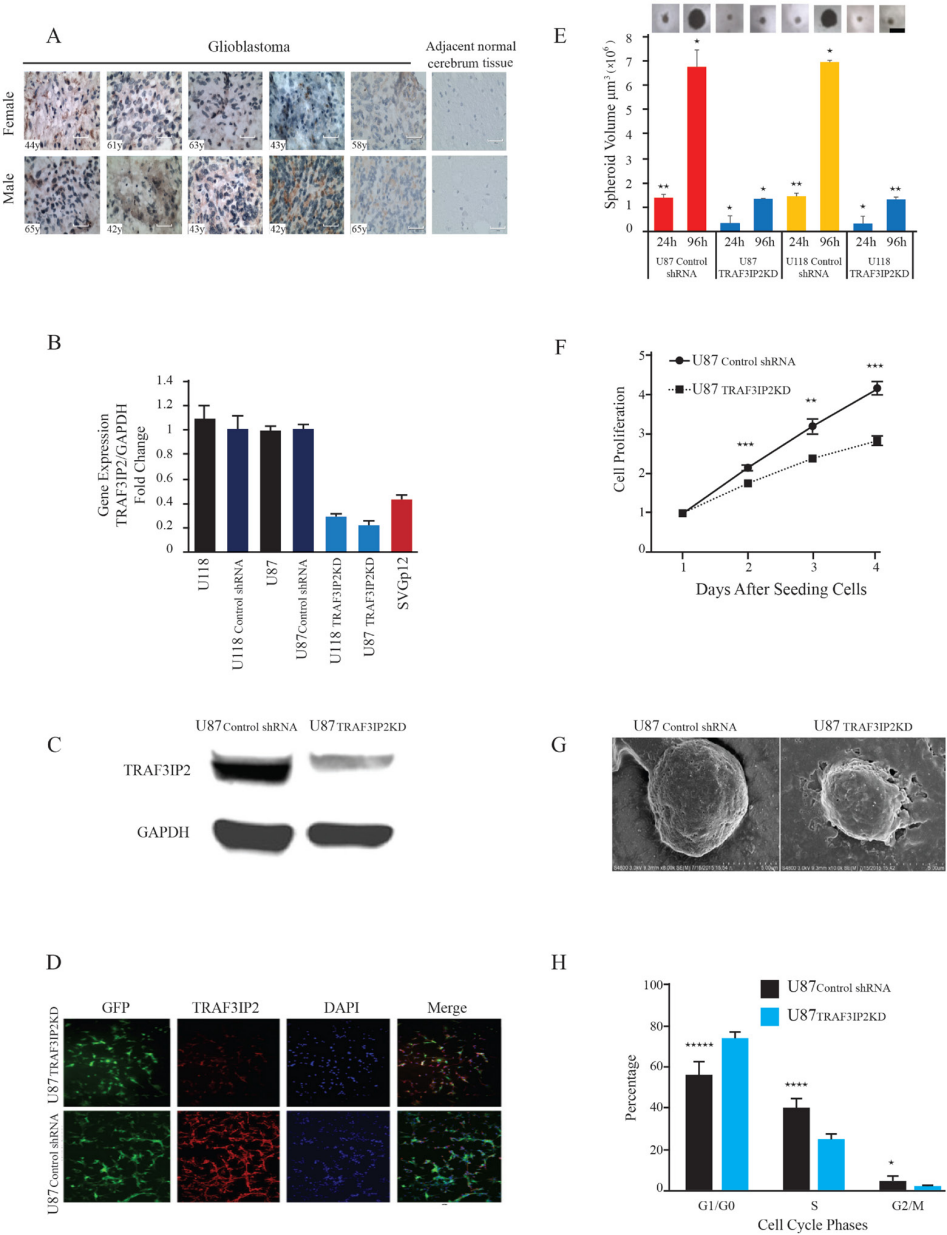
TRAF3IP2 expression in human glioblastoma tumor tissues and glioblastoma cell lines (**A**) TRAF3IP2 expression (brown) was localized by IHC. Hematoxylin was used as a counterstain (blue). Images representing glioblastoma tumor tissues from ten independent subjects are shown (5 females and 5 males, age of each subject is indicated on the image). The right panels show the images representing lack of TRAF3IP2 expression in adjacent non-tumor tissues. Scale bar, 100 μm. (**B**) TRAF3IP2 knockdown in U87 and U118 cells. TRAF3IP2 mRNA expression in U118, U118_control shRNA_, U87, U87_control shRNA_, U118_TRAF3IP2 KD_, U87_TRAF3IP2 KD_, and SVG p12 cells was analyzed by RT-qPCR. Results were normalized to values obtained in U87 and U118 cells respectively (*n* = 9/cell type; *P* < 0.05). (**C**) Western blot analysis of TRAF3IP2 expression in U87_TRAF3IP2 KD_ and U87_control shRNA_ cells. (**D**) Immunofluorescent detection of GFP (green) and TRAF3IP2 (red) in U87_TRAF3IP2 KD_ (top panels) and U87_control shRNA_ cells (bottom panels), counterstained with DAPI (blue) to visualize nuclei. Scale bar, 100 µm. (**E**) Effect of silencing TRAF3IP2 on sphere forming ability of U87_TRAF3IP2 KD_, U118_TRAF3IP2 KD_, U87_control shRNA_, U118_control shRNA_. Cells were incubated in sphere media for up to 96 hours. 20 spheroids/cell type were randomly selected for measurement at 24 and 96h time points. The spheres were imaged using a Nikon microscope. Spheroid diameters were measured using a microscope, and volumes computed (^*^P < 0.05; ^**^P < 0.01). (**F**) Analysis of U87_TRAF3IP2 KD_ and U87_control shRNA_ cell proliferation by XTT assay. Statistically significant differences at every time point; ^**^P < 0.01; ^***^P < 0.001. (**G**) Silencing TRAF3IP2 alters cell morphology. Morphology of U87_TRAF3IP2 KD_ and U87_control shRNA_ cells analyzed by uranyl acetate staining and viewed under electron microscopy (scale bar represents 500 nm). (**H**) Silencing TRAF3IP2 alters cell cycle profile. Mean and SEM of relative numbers of cells in G0/G1, S-Phase and G2/M phase of U87_TRAF3IP2 KD_ and U87_control shRNA_ cells (^*^P < 0.05; ^***^P < 0.001; ^****^P < 0.0001, *n* = 18).

In order to demonstrate the clinical relevance of TRAF3IP2 in glioblastoma, we analyzed its expression by IHC using a commercially available brain glioblastoma tissue array that contains tissue sections from 10 different glioblastoma patients of both genders and different ages. Adjacent normal brain tissue served as a control. The results showed a marked increase in TRAF3IP2 expression in all 10 cases of glioblastoma tumors (Figure 1A), compared to low or undetectable levels in adjacent normal brain tissue (*n* = 11) (Figure 1A), indicating that glioblastoma tumors express high levels of TRAF3IP2.

Similar to glioblastoma tumors (Figure 1A), the malignant U87 and U118 cells also expressed high levels of TRAF3IP2 mRNA (*versus* SVG p12 cells; 69.8%, *P<0.01*; Figure 1B). Further, lentiviral-mediated TRAF3IP2 shRNA decreased TRAF3IP2 expression by a significant 91.5% in both cell types (U87_TRAF3IP2KD_ *versus* U87_control shRNA_ and U118_TRAF3IP2KD_ *versus* U118_control shRNA_; both *P <* 0.0001; Figure 1B). Confirming RT-qPCR results, Western blotting demonstrated a significant ∼80% reduction in TRAF3IP2 protein levels in U87_TRAF3IP2KD_ cells (Figure 1C). Similarly, immunohistochemistry (IHC) confirmed a marked reduction in TRAF3IP2 levels in U87_TRAF3IP2KD_ cells (Figure 1D), demonstrating the efficacy of the shRNA used. However, the expression of gp130, used as an off-target, was not affected by the TRAF3IP2 shRNA (data not shown), demonstrating the specificity of the shRNA used.

It has been previously reported that a small subpopulation of tumors cells, characterized as cancer stem cells (CSCs), is able to form spheroids [23, 24]. Therefore, we investigated whether silencing TRAF3IP2 affects the sphere-forming ability of glioblastoma cells. Our data show that both wild type U87 and U118 cells display high sphere-forming ability during the 24- to 96-hour study period (Figure 1E), an effect markedly reduced by TRAF3IP2 knockdown (U87_TRAF3IP2KD_ and U118_TRAF3IP2KD_; 20 spheroids/cell type were randomly selected for measurement; triplicate experiments; *P<0.05*), demonstrating the role of TRAF3IP2 in sphere-forming potential of malignant glioblastoma cells.

Silencing TRAF3IP2 also inhibited proliferation of U87_TRAF3IP2KD_ cells (*versus* U87_control shRNA_ cells) during the 4-day study period (Figure 1F). Targeting TRAF3IP2 also modified the ultrastructure of U87 cells (U87_TRAF3IP2KD_) (Figure 1G). Further, TRAF3IP2-silenced cells showed a significant increase in the number of cells in G0/G1 phase (*P* < 0.001) and a marked decrease in S and G2 phases (*P <* 0.05), indicating that silencing TRAF3IP2 inhibits proliferation of malignant U87 glioblastoma cells (Figure 1H).

### Genome profiling of messenger RNA in TRAF3IP2-silenced U87 glioblastoma cells

**Figure 2:**
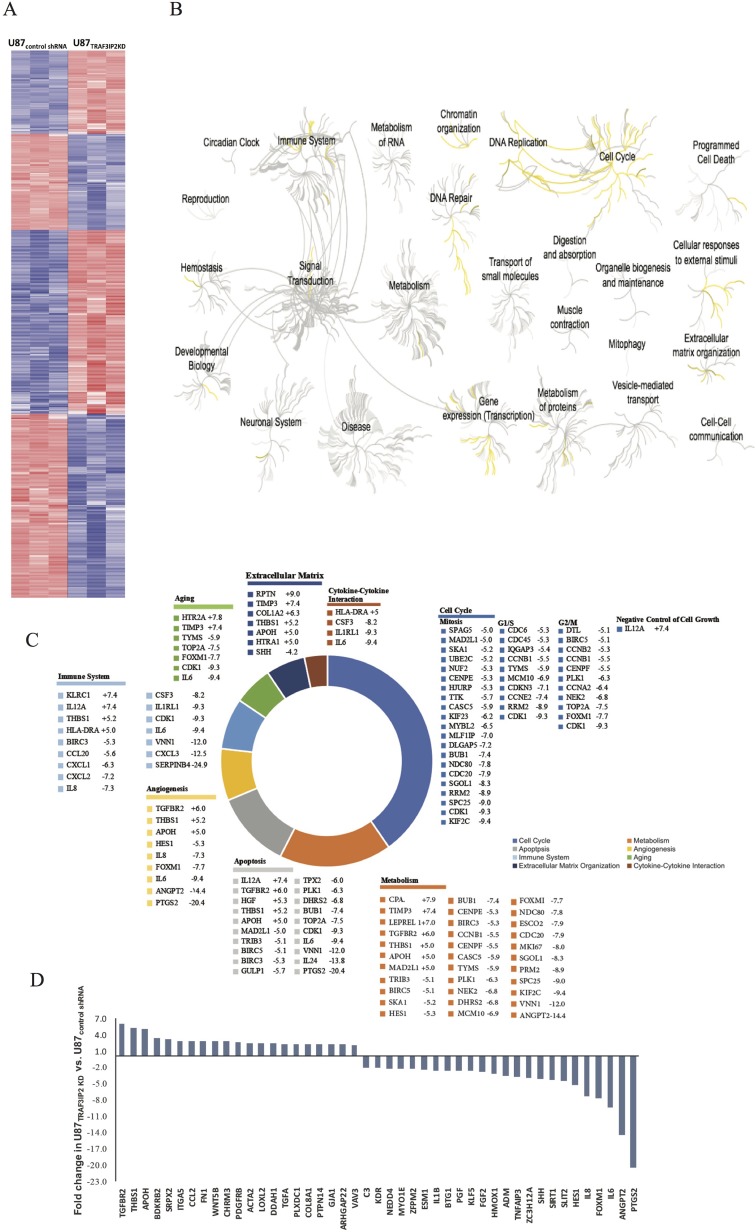
Differential gene expression in U87_TRAF3IP2 KD_ and U87_control shRNA_ cells (**A**) Hierarchical clustering displayed genes differentially expressed in U87_TRAF3IP2 KD_ and U87_control shRNA_ cells. The extent of blue (decreased fold change) or red (increased fold change) color is directly proportional to the magnitude of differential expression of these genes. (**B**) Reactome (http://reactome.org/) was used for Gene Ontology Tree, representing functional characterization of genes differentially expressed in U87_TRAF3IP2 KD_ cells and U87_control shRNA_ cells in comparition to the number (and significance) of gene ontologies and shows a dendrogram comparison of gene ontology (biological process) specifically differential between U87_TRAF3IP2 KD_ cells and U87_control shRNA_ cells. Yellow, ontologies enriched in U87_TRAF3IP2 KD_ cells; gray, ontologies not affected in U87_TRAF3IP2 KD_ cells. Inset, the top four biological processes hit. Of particular interest is the specific and significant enrichment of proteins involved in cell cycle, DNA replication, immune system, programmed cell death, cellular responses to external stimuli, extracellular matric organization, DNA repair and metabolism. (**C**) Pathway analysis (using Reactome) of a cluster of 1297 perturbed gene expressions in U87_TRAF3IP2 KD_ cells revealed a statistically significant preponderance of genes involved in cell cycle, metabolism, apoptosis, angiogenesis, immune system, aging, extracellular matrix organization, and cytokine-cytokine interaction. The chart displays genes representative of each pathway displaying greater than 5-fold change in U87_TRAF3IP2 KD_ versus U87_control shRNA_ cells (*P* < 0.05). (**D**) Fold change expression of perturbed genes involved in angiogenesis in U87_TRAF3IP2 KD_ versus U87_control shRNA_ cells (*P* < 0.05).

Figure 2A shows hierarchical clustering of differentially expressed genes in TRAF3IP2-silenced U87 cells compared to control shRNA-transfected cells (U87_TRAF3IP2KD_ *versus* U87_control shRNA_ cells). The data show that the expression of 1,297 genes was significantly perturbed in U87_TRAF3IP2KD_ cells, of which 597 were significantly upregulated (>2-fold) and 700 downregulated. Further, gene ontology analysis revealed that silencing TRAF3IP2 significantly affects the expression of genes involved in cell cycle progression, immune activation, cytokine-cytokine interaction, aging, apoptosis, extracellular matrix organization, DNA replication, repair and metabolism (Figure 2B). A significant alteration was also observed in canonical pathways related to angiogenesis in U87_TRAF3IP2KD_ cells (*versus* U87_control shRNA_ cells; Figure 2C; Supplementary Tables 1–4).

Expression of Prostaglandin-Endoperoxide Synthase-2 (PTGS2, also known as COX-2) has been shown increased in various tumors, including glioblastoma [25]. Notably, PTGS2 expression was downregulated by a significant ∼20-fold in U87_TRAF3IP2KD_ cells (Figure 2D; Supplementary Tables 1–4). Expression levels of Cyclin-Dependent Kinase (CDK-1, CDK-2, and CDK-6) were also significantly down-regulated in U87_TRAF3IP2KD_ cells (-9.3, -2.4, and -2.1, respectively; Supplementary Table 1), indicating that silencing TRAF3IP2 affects glioblastoma cell cycle progression.

### Silencing TRAF3IP2 inhibits NF-κB activation and proinflammatory/pro-angiogenic mediators in malignant U87 glioblastoma cells

**Figure 3:**
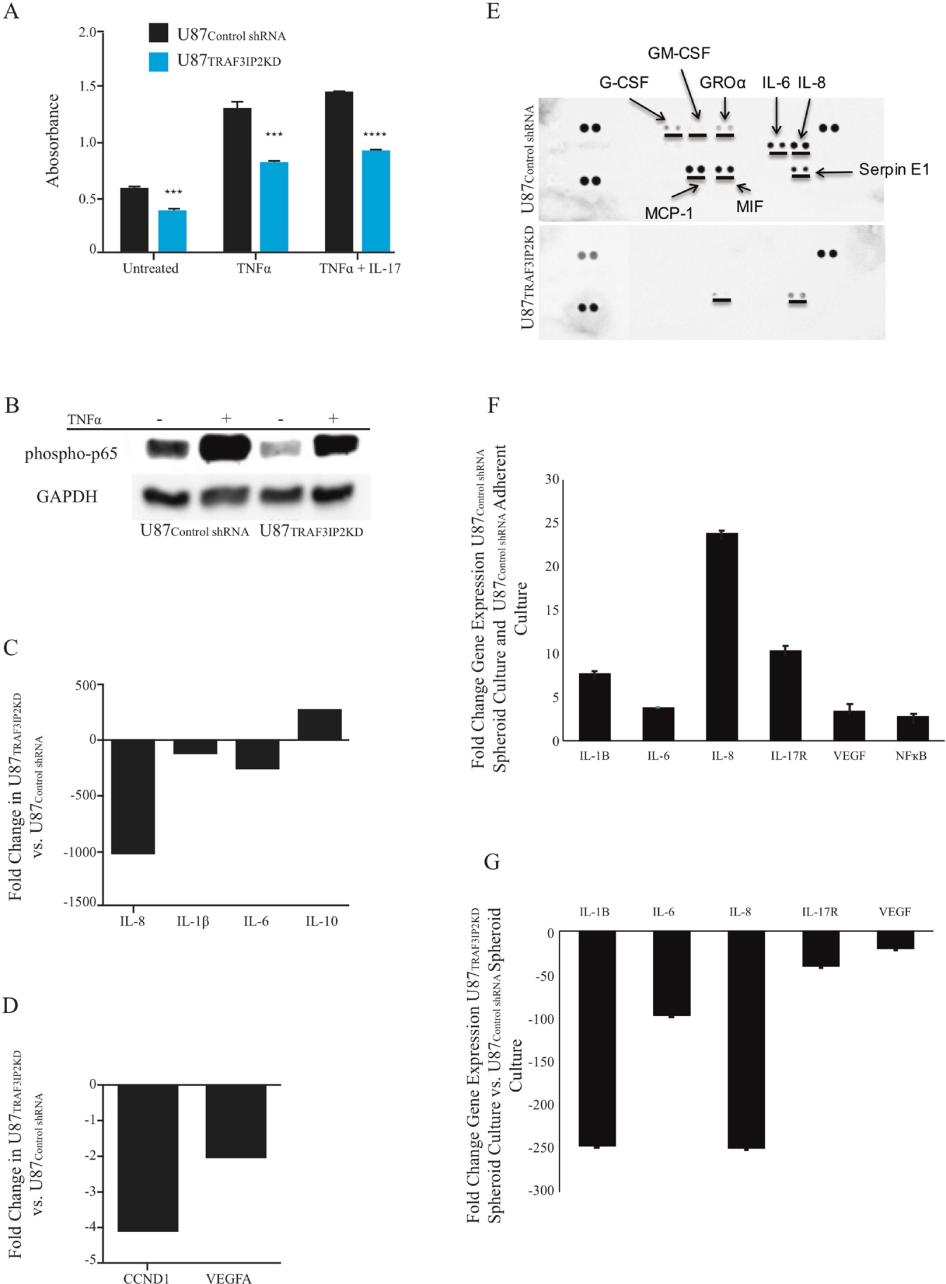
Silencing TRAF3IP2 inhibits NF-κB activation and inflammatory cytokine expression in malignant glioblastoma cells (**A**) p-p65 levels were analyzed by ELISA. Silencing TRAF3IP2 inhibits TNF-α or TNF-α+IL-17-induced p-p65 levels in U87_TRAF3IP2KD_ and U87_control shRNA_ cells. (**B**) Western blot analysis demonstrating significantly reduced p-p65 levels in U87_TRAF3IP2 KD_ versus U87_control shRNA_ cells. U87_TRAF3IP2 KD_ and U87_control shRNA_ cells showed higher expression of p-p65 after TNF-α treatment. However, the magnitude of increase is less in U87_TRAF3IP2KD_ cells. (**C** and **D**) RT^2^-qPCR analysis: fold changes of U87_TRAF3IP2KD_ versus U87_control shRNA_ cells for IL-8, IL-1β, IL-6, IL-10, CCND1 and VEGF. (**E**) Dot-blot comparative protein analysis of conditioned media from U87_TRAF3IP2KD_ cells showing decreased expression of G-CSF, GM-CSF, GRO, IL-6, IL-8, MCP-1 and MIF compared to U87_control shRNA_ cells. Protein levels of G-CSF, GM-CSF, GRO, IL-6, IL-8 and MCP-1 were below the detection limit in U87_TRAF3IP2KD_ cells conditioned media. (**F**) RT-qPCR analysis: fold changes expression of upstream (IL-17R) and downstream (NF-κB, IL-1β, IL-6, and IL-8, in addition to VEGF) signaling of TRAF3IP2 in U87 spheroids compared to adherent U87 cultures. (**G**) Gene expression analysis of U87_TRAF3IP2KD_ versus U87_control shRNA_ spheroids (*n* = 6; ^*^P < 0.05, ^**^P < 0.001, ^***^P < 0.0001).

The data in Figure 3A show that silencing TRAF3IP2 not only reduced basal (34.5% in U87_TRAF3IP2KD_ *versus* U87_control shRNA_ cells), but also TNF-α+IL-17-induced NF-κB activation (37.4%), as evidenced by a significant reduction in p-p65 levels. Western blot analysis confirmed these ELISA findings and showed that while silencing TRAF3IP2 decrease p-p65 level by ∼40% in U87_TRAF3IP2KD_ compared to U87_control shRNA_ cells, TNF-α -induced increases in p-p65 by ∼30% (Figure 3B). Further, silencing TRAF3IP2 also inhibited proinflammatory IL-8, IL-6, and IL-1β expression, with a concomitant increase in IL-10 expression (*versus* U87_control shRNA_ cells; Figure 3C). Silencing TRAF3IP2 also decreased the expression of Cyclin D1, a cell cycle regulator, by 4-fold and that of VEGF by 2-fold (*P* < 0.0001; Figure 3D). Proteomic profiling of conditioned medium from U87_TRAF3IP2KD_ cell cultures demonstrated a marked decrease in secreted IL-8, IL-6 and IL-1β levels (*versus* U87_control shRNA_ cells; *P* < 0.01; Figure 3E). However, levels of MCP-1, MIF, G-CSF, GM-CSF, and GROα were below detection level, and that of SERPIN E1 (PAI-1) did not significantly change. These results indicate that targeting TRAF3IP2 inhibits various mediators involved in inflammation, proliferation, and cell cycle progression.

Our data also show that, compared to adherent wild type U87 cultures, free-floating spheroids formed by these cells expressed high levels of pro-inflammatory cytokines (Figure 3F). However, the spheroids formed by TRAF3IP2-silenced U87 cells (U87_TRAF3IP2KD_ *versus* U87_control shRNA cells_) expressed markedly reduced VEGF, IL-17R, IL-1β, IL-6, and IL-8 expression (Figure 3G), further demonstrating a pro-angiogenic and pro-inflammatory role of TRAF3IP2 in glioblastoma cells.

### Silencing TRAF3IP2 blunts the tumorigenic potential of U87 glioblastoma cells

**Figure 4:**
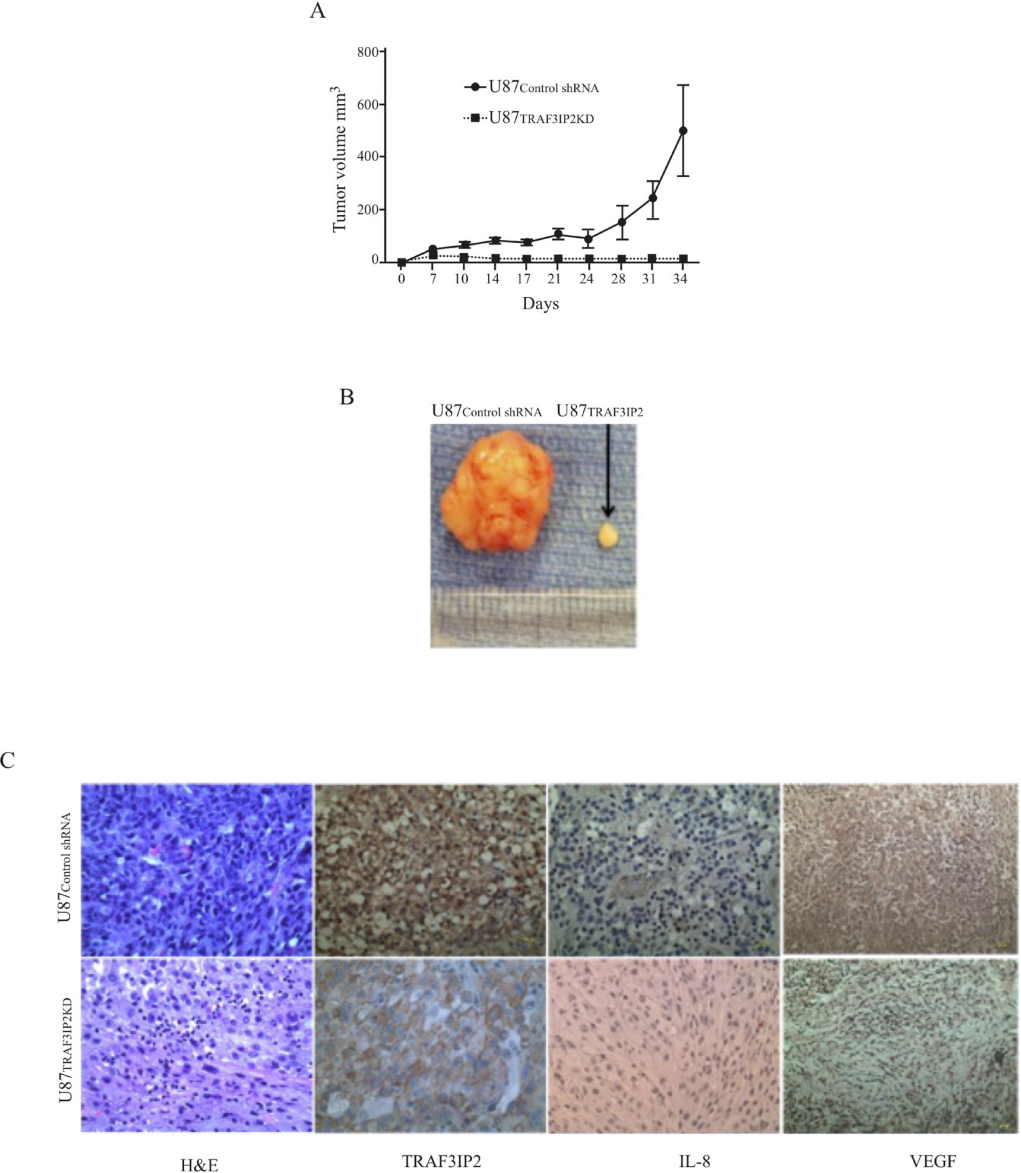
Silencing TRAF3IP2 prevents glioblastoma growth (**A**) Immunodeficient NIH-III mice were injected with U87_TRAF3IP2KD_ cells (1 × 10^6^ cells) into the flank region. Control animals were injected with U87_control shRNA_ cells (1 × 10^6^ cells). Tumor size was measured weekly using calipers. (**B**) U87_TRAF3IP2KD_ cells formed smaller tumors. (**C**) Immunohistochemical localization of TRAF3IP2, IL-8, and VEGF in tumors derived from U87_TRAF3IP2KD_ and U87_control shRNA_ cells. Scale: 100 µm.

To investigate the role of TRAF3IP2 in the tumorigenicity of malignant U87 glioblastoma cells, we injected TRAF3IP2-silenced U87 cells (U87_TRAF3IP2KD_ cells) into the flank region of immunodeficient NIH-III mice and followed for up to 60 days. The data showed that U87 cells traduced with scrambled shRNA (U87_control shRNA_) formed markedly larger tumors earlier compared to U87_TRAF3IP2KD_ cells (10.8 mg *versus* 1790.8 mg; *P* < 0.0001; Figure 4A and 4B). Intriguingly, following an initial linear growth during the first 10 days, the tumors formed by U87_TRAF3IP2KD_ cells even regressed over the next 50-day study period (*P* < 0.01). Further analysis of residual tumors revealed significantly low levels of TRAF3IP2, IL-8, and VEGF expression (Figure 4C). These data indicate that targeting TRAF3IP2 inhibits glioblastoma growth.

### Therapeutic significance of targeting TRAF3IP2 in the regression of pre-existing glioblastoma tumors

**Figure 5:**
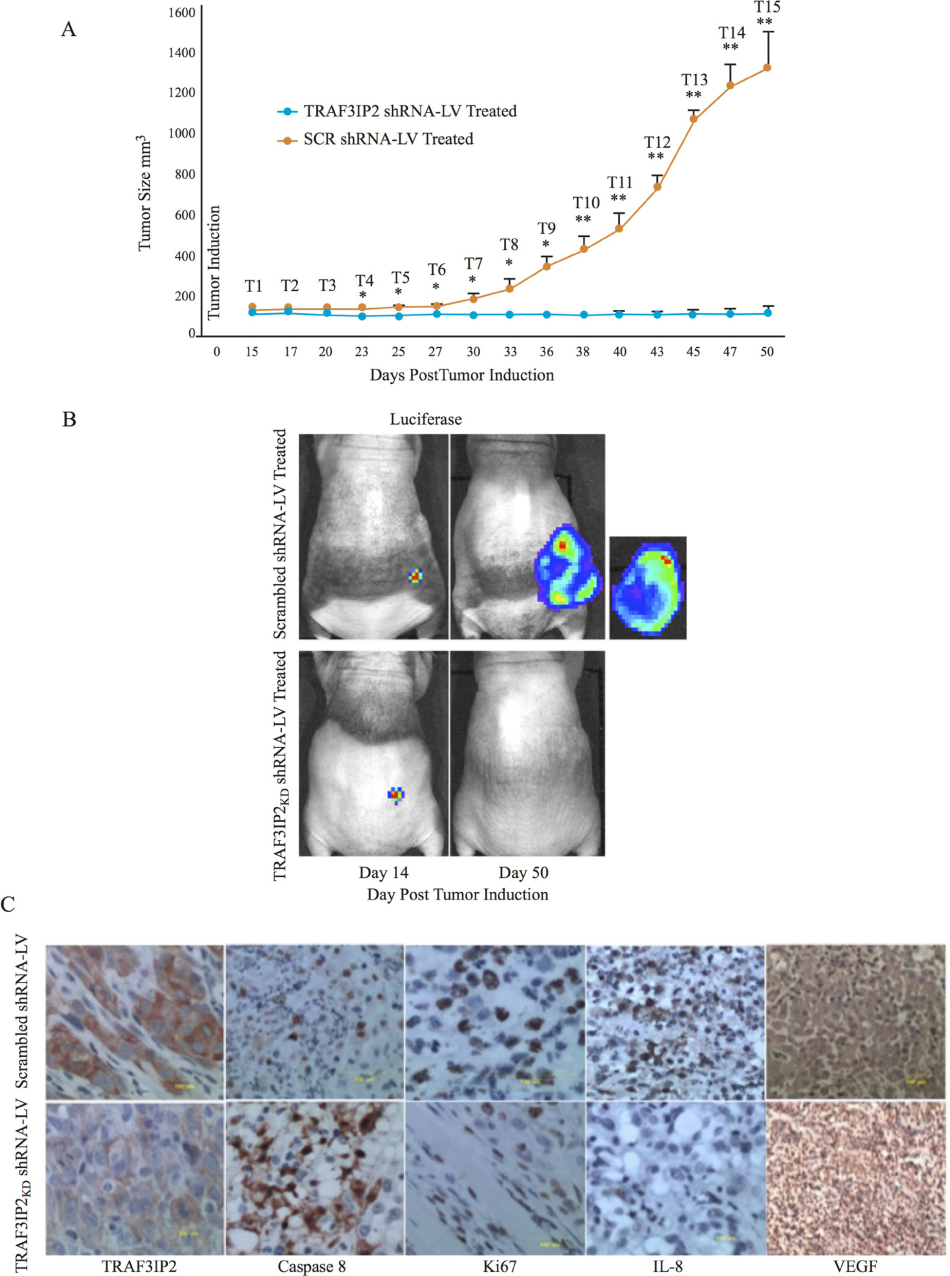
Effect of silencing TRAF3IP2 in a flank xenograft model (**A**) Suppression of glioblastoma tumors by TRAF3IP2 shRNA-LV injected subcutaneously onto tumors compared to scrambled shRNA-LV injected tumors. Frequency of administration is shown in the graph. (**B**) Tumor size was measured biweekly (^*^P < 0.05; ^**^P < 0.001). (**C**) Animals imaged for luciferase weekly. Immunohistochemical localization of TRAF3IP2, caspase 8, Ki67, IL-8, and VEGF in tumors treated with TRAF3IP2 shRNA-LV or scrambled shRNA-LV. Scale: 100 µm.

Having demonstrated that TRAF3IP2-silenced malignant U87 glioblastoma cells form significantly smaller tumors, we next determined whether treating existing tumors by lentiviral TRAF3IP2 shRNA regresses their size. In this translationally important strategy, tumors were induced at first by injecting luciferase-labeled U87 cells into the flank region of immunodeficient NIH-III mice. Fourteen days later, when tumors were distinctively quantifiable, lentivirus expressing GFP-tagged TRAF3IP2 shRNA (TRAF3IP2 shRNA-LV) was injected subcutaneously onto the tumors. Scrambled shRNA-LV served as a control. Results in Figure 5A show a remarkable reduction in tumor size over 50 days post-induction in TRAF3IP2 shRNA-LV-treated animals (*versus* scrambled shRNA-LV; 0.08 ± .03 g *versus* 1380 ± 48, respectively) (Figure 5B). Analysis of residual tumors by IHC revealed a marked reduction in TRAF3IP2, Ki67, IL-8 and VEGF expression (Figure 5C), but a significant increase in caspase 8 levels (Figure 5C).

**Figure 6:**
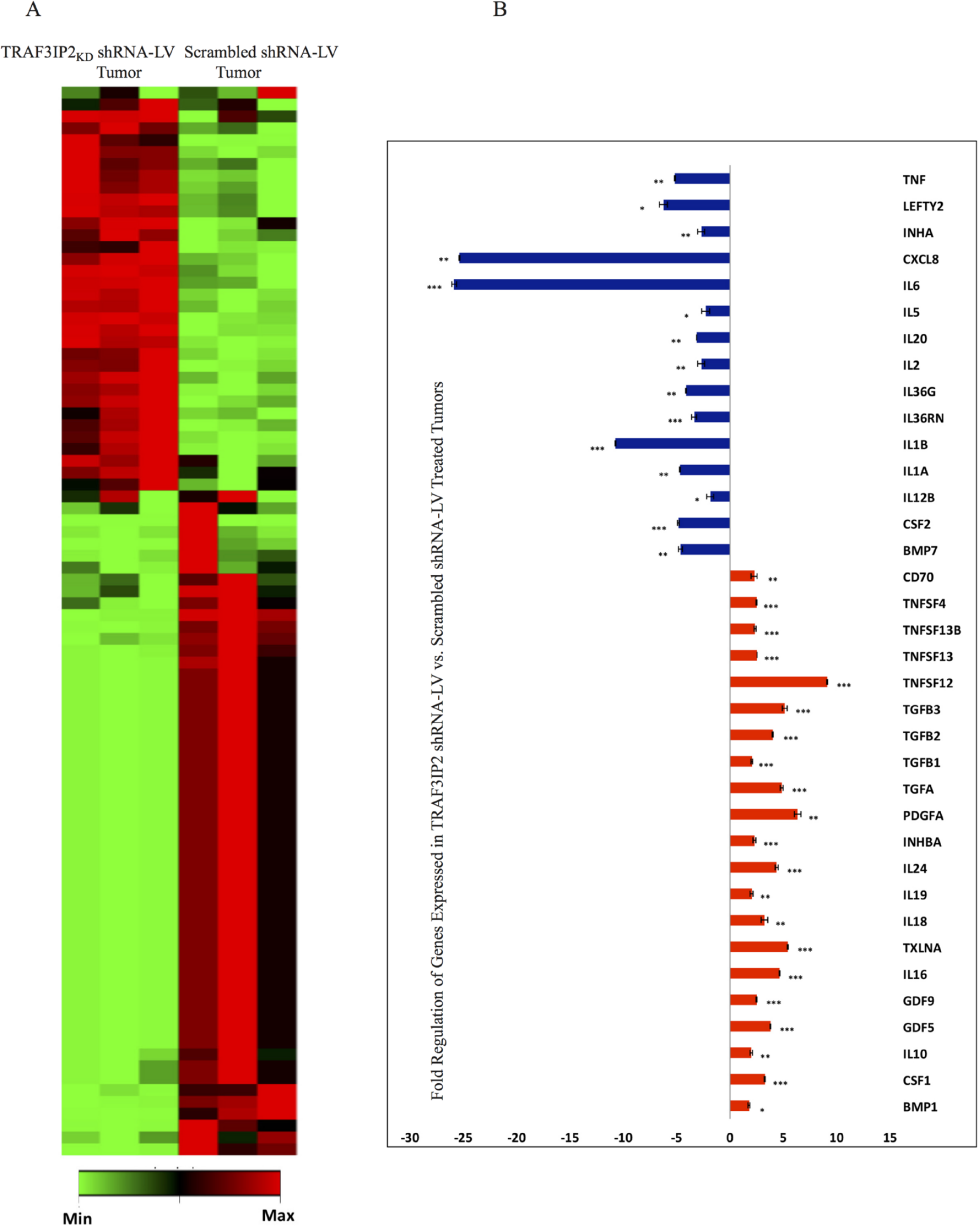
Transcriptome analysis of TRAF3IP2 shRNA-LV-treated xenograft tumors (**A**) Clustergram of genes analyzed by RT^2^-based PCR array. Xenograft glioblastoma tumors treated with TRAF3IP2 shRNA-LV were compared with scrambled shRNA-LV treated tumors (*n* = 3/group). (**B**) Clustering performed using data analysis software (Qiagen). Intensity of green (decreased fold change) or red (increased fold change) is directly proportional to the magnitude of differentially expressed genes. Expression of genes displaying a +/− 2-fold change in TRAF3IP2 shRNA-LV-treated xenograft tumors. Values normalized to scrambled shRNA-LV treated tumors (^*^P < 0.05; ^**^P < 0.01; ^***^P < 0.001).

RT^2^-based transcriptome analysis revealed that a cluster of 36 genes was differentially expressed with a minimum of 2-fold change (Figure 6A). Interestingly, similar to the results obtained in *in vitro* studies (Figure 3D and 3E), the expression of IL-8, IL-6 and IL-1β mRNA was significantly inhibited in TRAF3IP2 shRNA-LV-treated tumors, while the expression IL-10 mRNA was markedly up regulated (Figure 6B). These results demonstrate that targeting TRAF3IP2 inhibits tumor growth by suppressing inflammatory cytokine expression.

## DISCUSSION

**Figure 7:**
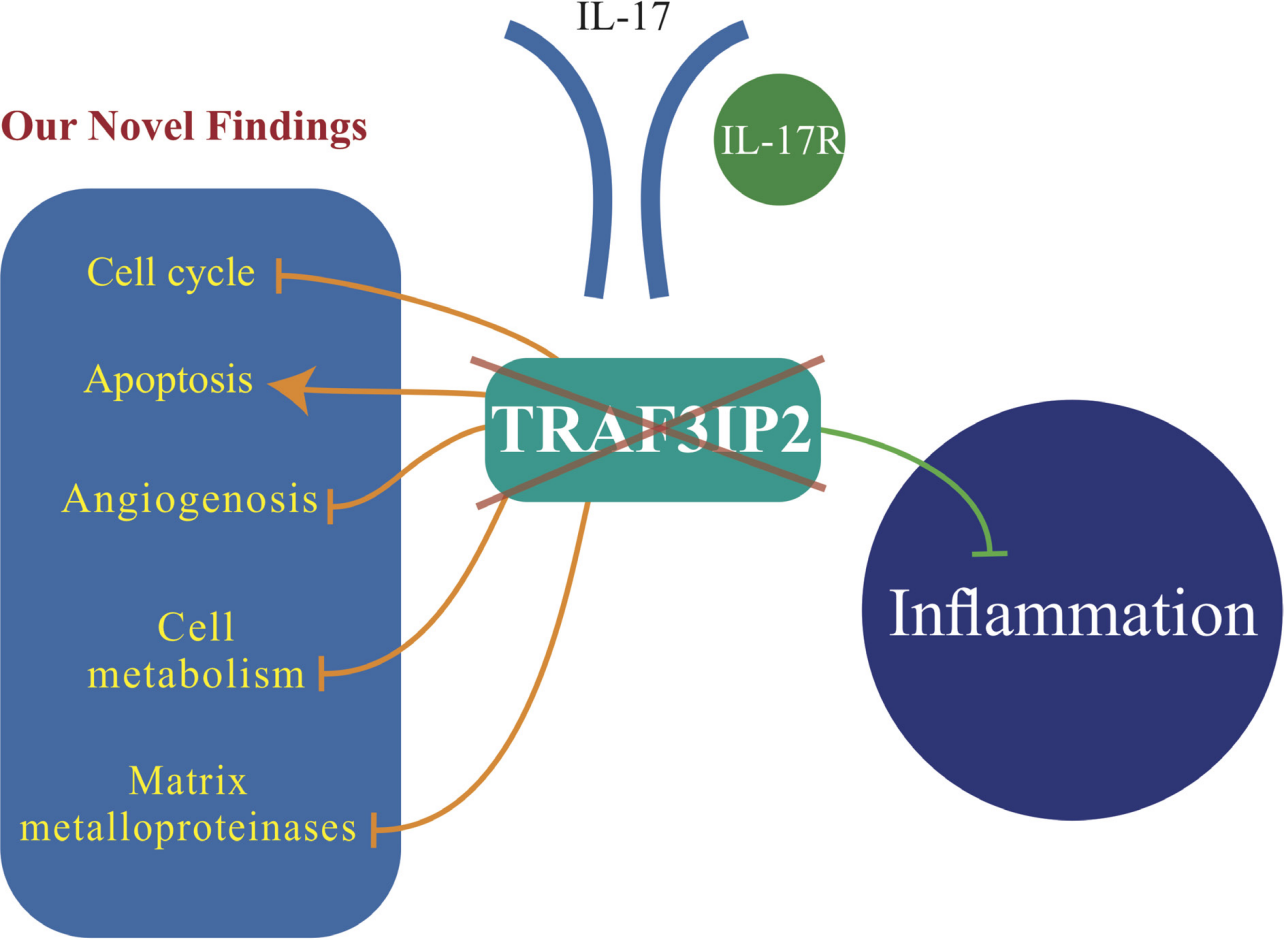
TRAF3IP2 is a potential therapeutic target in glioblastoma growth and dissemination In addition to blocking inflammation (green line), our novel findings show that silencing TRAF3IP2 inhibits cell cycle progression, angiogenesis, cell metabolism, and matrix metalloproteinase expression, while increasing apoptosis of glioblastoma cells (orange lines), resulting ultimately in tumor regression, and possibly elimination.

Here we report several novel findings: 1. Primary human glioblastoma tumors express high levels of TRAF3IP2 (Figure 1); 2. Silencing TRAF3IP2 markedly inhibits the sphere forming potential of malignant U87 glioblastoma cells under serum-free conditions (Figure 1); 3. Silencing TRAF3IP2 suppresses activation of NF-κB and induction of pro-inflammatory mediators, as well as mediators involved in cell cycle and metabolism in U87 glioblastoma cells (Figures 2 and 3); 4. Wild type U87 glioblastoma cells form large tumors in the flank xenograft model and express high levels of TRAF3IP2 (Figure 4); 5. TRAF3IP2-silenced U87 glioblastoma cells form significantly smaller tumors in the flank xenograft model (Figure 4); and 6. Treating existing tumors formed by the wild type U87 glioblastoma cells with TRAF3IP2 shRNA significantly reduces tumor size in the flank xenograft model (Figure 5). 7. More importantly, targeting TRAF3IP2 suppresses the expression of various pro-angiogenic mediators, including VEGF. Concertedly, these *in vitro* and *in vivo* data identify TRAF3IP2 as a novel and a potential therapeutic target in glioblastoma (Figure 7).

In glioblastoma, the TME consists of both cellular and soluble compartments. The soluble compartment consists of pro-tumorigenic inflammatory mediators, such as cytokines and chemokines that play a vital role in the communication between malignant and non-malignant cells, resulting in glioblastoma growth and dissemination. Importantly, silencing TRAF3IP2 made the TME less inflammatory and tumorigenic, revealing its crucial role in pro-inflammatory and pro-tumorigenic TME in glioblastoma.

Our data also show for the first time that silencing TRAF3IP2 inhibits the spheroid forming ability of malignant glioblastoma cell lines U87 and U118. It has been previously reported that gliomas and other primary brain tumors contain a sub-population of cells that express stem cell-like properties (cancer stem cells or CSCs) [26], and contribute to tumor growth and drug resistance, and possibly contributing to tumor recurrence. In other words, though current treatments reduce overall tumor mass, cessation of treatment results in recurrence of tumors due to the presence of drug-resistant CSCs in the tumor, that again grow to form tumors that display higher resistance to intervention. This reduces the efficacy and effectiveness of subsequent rounds of treatment. Our novel data show that silencing TRAF3IP2 inhibits the spheroid-forming ability of malignant glioblastoma cell lines, indicating that targeting TRAF3IP2 not only inhibits the proliferative potential of tumor cells, but also the growth of CSCs, ultimately eliminating the tumor and its recurrence.

The physiological importance of TRAF3IP2 is reflected in its ability to regulate multiple signaling pathways downstream of the IL-17R and contribute to a variety of inflammatory pathologies [27, 28]. In fact, TRAF3IP2 resides in a known susceptibility locus for inflammatory diseases such as psoriasis and psoriatic arthritis, Crohn’s disease, and type I diabetes [29–31]. Several reports have demonstrated that TRAF3IP2 is an upstream regulator of NF-κB activation in various cell types, including glioma cells [32, 33]. In this study, we have shown that TRAF3IP2 is overexpressed in malignant U87 glioblastoma cells, and its silencing inhibits both basal and inducible NF-κB activation. It has been previously reported that targeting NF-κB activation or NF-κB-dependent gene expression reduces brain tumor growth, angiogenesis and invasion [34]. Thus, there is a strong correlation between persistent NF-κB activation and gliomagenesis. Also, it has been shown that TLR4 signaling culminates in the activation of pro-inflammatory NF-κB and the promotion of a pro-inflammatory TME [35], suggesting that chronic NF-κB activation is detrimental and exerts pro-inflammatory and pro-tumorigenic effects. Our data demonstrated that targeting TRAF3IP2 inhibits NF-κB activation and pro-inflammatory TME, possibly contributing to tumor regression.

Our data also show that targeting TRAF3IP2 expression in malignant U87 cells perturbs various genes involved in cell cycle regulation (170 genes), apoptosis (124 genes), angiogenesis (45 genes), immune system (72 genes), and extracellular matrix organization (45 genes). For example, the expression of PTGS2, also known as COX-2, showed a significant ∼21-fold decrease in U87_TRAF3IP2KD_ cells. Also, the expression of genes involved in cell cycle regulation, such as CDK-4 and CDK-6, is significantly reduced in TRAF3IP2-silenced cells. Cyclins regulate CDK kinases, and different cyclins exhibit distinct expression and degradation patterns and contribute to temporal coordination of each mitotic event. The inhibitors of CDK-4 and CDK-6 have shown promise in glioblastoma treatment [36]. In addition, PTGS2 upregulation is demonstrated to modulate tumor growth by regulating apoptosis and angiogenesis in glioblastoma [37]. Increased PTGS2 expression also confers multidrug resistance. Interestingly, IL-1β induces PTGS2 expression in part via NF-κB and AP-1 activation. In contrast, silencing TRAF3IP2 upregulates the mRNA expression of IL-10, an anti-inflammatory cytokine in U87 cells *in vitro*. More importantly, targeting TRAF3IP2 inhibits various mediators involved in angiogenesis, including VEGF. Together, these results suggest that silencing TRAF3IP2 favors a less inflammatory and a less tumorigenic TME, and reduced angiogenesis, possibly contributing to smaller tumors and regression.

In glioblastoma, several genes involved in tumor cell survival are investigated as potential therapeutic targets. For example, the expression of KIF2C, which promotes mitotic chromosome segregation, is considerably upregulated in glioblastoma [38]. Our data show that silencing TRAF3IP2 significantly inhibits its expression in glioblastoma cells. Further, kinensins, such as EG5 (also known as KIF1 for Kinensin Family member 11) and CENPE (Centromere-associated Protein E) are being targeted in ongoing Phase I and II clinical trials [39]. Notably, our data show that silencing TRAF3IP2 decreases the expression of both EG5 and CENPE. IL-12 is a major activator of both innate (NK cells) and adaptive (cytotoxic T lymphocytes) immunity, and is being considered an ideal candidate for tumor immunotherapy. In fact, in a recently initiated clinical trial, IL-12 is being tested as an adjuvant in cancer vaccines and a tumor-targeting immunocytokine [40]. Our data show that silencing TRAF3IP2 significantly increased its expression in U87 glioblastoma cells, possibly contributing to reduced tumor growth in the flank xenograft model. Though the nude mice are immunodeficient, these data indicate that silencing TRAF3IP2 inhibits the pro-tumorigenic KIF2C, EG5 and CENPE, but upregulates IL-12 mRNA expression, resulting ultimately in smaller tumors that did not further grow.

Another novel finding of our study is that silencing TRAF3IP2 inhibits the expression of both SERPINB4 and SERPIN2 in the U87 glioblastoma cell line. SERPINB4 is expressed at high levels in many tumor cells, and is known to inactivate granzyme M, an enzyme involved in tumor cell death. It has been identified that oncogenic RAS protein upregulates SERPINB3/4 and subsequent cytokine production and tumor growth [41, 42]. The DNA topoisomerase TopIIα along with reactive oxygen species plays an important role in genomic DNA damage and random integration. It has previously been shown to serve as a prognostic factor in glioblastoma patients [43, 44]. Importantly, the DNA alkylating agent temozolomide, which is used in combination with the standard post-operative radiotherapy, is known to target TopIIα expression [43]. Our data show that silencing TRAF3IP2 down regulates TOP2A expression by a significant ∼7.5-fold in the malignant U87 glioblastoma cell line.

These proof-of-principal studies using human malignant glioblastoma cell lines show the crucial role of TRAF3IP2 in GBM pathology. Our data show that primary human glioblastoma tumors and malignant glioblastoma cell lines express high levels of TRAF3IP2, and targeting its increased expression blunts various pathways critical to development and progression of tumors, including angiogenesis, cell cycle progression, cell metabolism, and matrix metalloproteinase expression. Interestingly, several gene products that are being targeted in ongoing clinical trials are TRAF3IP2-responsive genes, indicating that TRAF3IP2 could be a potential and promising therapeutic target in glioblastoma, a fatal disease.

### Limitations and future directions

The study aimed to investigate the causal role of TRAF3IP2 in glioblastoma pathology. Our data showed high levels of TRAF3IP2 expression in primary human glioblastoma tumors. Supporting these observations, the human malignant glioblastoma cell lines U87 and U118 also expressed high levels of TRAF3IP2, justifying the use of these cell lines in the current study. As a proof-of-concept, we used the flank xenograft model to study the causal role of TRAF3IP2 and its potential as a therapeutic target in glioblastoma. In fact, several glioblastoma milestone studies used the flank xenograft model to identify novel therapeutics and therapeutic targets. Our future studies will determine the causal role of TRAF3IP2 in the pathogenesis of glioblastoma using the brain PDX (patient-derived xenograft) model, which is the host tissue for glioblastoma.

## MATERIALS AND METHODS

### Culture of malignant glioblastoma cell lines, non-malignant glial cells and spheres

Human malignant glioblastoma cell lines U87 and U118, and non-malignant SVG p12 were purchased from ATCC (Rockville, MD, USA). U87 and U118 cell lines were derived from 44 and 50 year-old glioblastoma patients, respectively. SVG p12 is a astroglial cell line originated from first trimester fetal brain and subsequently transformed with SV40. The vendor authenticated all cell lines for sterility (mycoplasma, aerobic and anaerobic), and declared free of pathogens (PCR-based assay for HIV, HepB, HPV, EBV, and CMV). While U87 and U118 cells display epithelial-like morphology, the SVG p12 cells show a fibroblastic morphology. We routinely verified their morphology under phase contrast microscope. Both U87 and U118 cells display a relatively high colony forming efficiency on agarose, indicating their transformation and tumorigenic potential. We monitored their tumorigenic potential by subcutaneous injection into the flank region (5 × 10^5^ cells/cell line in Matrigel) of immunodeficient nude mice every 6 months. The cells were cultured in Eagle’s Minimum Essential Medium containing 1% GlutaMAX, 100 U/ml penicillin/streptomycin and 10% FBS (all from Thermo Fisher Scientific) at 37° C in a humidified 5% CO_2_ incubator.

### Spheroids

For sphere formation, cultures of adherent U87_TRAF3IP2KD_, U118_TRAF3IP2KD_, U87_control shRNA_ and U118_control shRNA_ cells were switched to serum-free medium containing 20 ng/ml of basic fibroblast growth factor (Peprotech, Rocky Hill, NJ, USA), 20 ng/ml of epidermal growth factor (Peprotech) and 20 ng/ml leukemia inhibitory factor (Chemicon) in low attachment culture dishes. 20 spheroids/cell type were randomly selected for measurement at 24 and 96 h time points. A Nikon microscope (Nikon TE2000-U and Metamorph Image Analysis software) was used to measure diameters of spheroids, and the major (a) and minor (b) radius were calculated using the following formula:

$$V=\frac{4}{3}\pi a^{2}b$$

### Transduction of lentiviral particles

To silence/knockdown (KD) TRAF3IP2 expression, U87 and U118 cells were transduced with lentiviral TRAF3IP2-shRNA (MOI 1) (U87_TRAF3IP2KD,_ U118_TRAF3IP2KD_). Scrambled-shRNA (U87_control shRNA_ and U118_control shRNA_) and PBS served as controls. Polybrene^®^, a cationic polymer (Santa Cruz Biotechnology, Inc.) was used to increase transduction efficiency. Neither shRNA nor Polybrene^®^ affected cell viability or off-target expression (data not shown). The transduced population was selected using puromycin (500 ng/ml; Thermo Fisher Scientific), cloned at a single cell level, and the resulting colonies analyzed for TRAF3IP2 expression by RT-qPCR. The colony displaying the highest knockdown was selected and used in the present study.

### Immuncytochemistry/Immunofluorescence

Cells were fixed in 4% paraformaldehyde, permeabilized with Triton X-100, blocked, incubated overnight at 4° C with anti-TRAF3IP2 antibody (Santa Cruz Biotechnology, Inc.), washed and further incubated with a secondary antibody. Nuclei were stained with DAPI. Images were acquired with a Nikon TE2000-U microscope.

### Western blot analysis

Whole cell extracts were prepared using Cell Lysis Buffer supplemented with a Protease Inhibitor Cocktail. Equal amounts of cell lysates were analyzed for TRAF3IP2 (Santa Cruz Biotechnology, Inc.) and phospho-p65 (p-p65, Ser536, Cell Signaling Technology, Inc.) expression by western blotting. Immuno-reactive bands were detected using Signal Fire Plus ECL Reagent and quantified (Image Quant LAS4000, GE Healthcare Life Sciences).

### Cell proliferation assay

U87 cells were seeded into 96-well plates at a density of 1 × 10^4^/well. After 14 hours, 50 μl of 2,3-Bis-(2-Methoxy-4-Nitro-5-Sulfophenyl)-2*H*-Tetrazolium-5-Carboxanilide assay (XTT) detection solution (Cell Signaling Technology, Inc.) was added and analyzed at 450 nm and 630 nm on each day for 4 consecutive days with a spectrophotometer (*n* = 3). Cell proliferation was calculated according to the following formula:

$$\frac{\left(\mathrm{OD}450\mathrm{nm}-\mathrm{OD}630\mathrm{nm}\right)}{\left(\mathrm{OD}450\mathrm{nm}-\mathrm{OD}630\mathrm{nm}\right)\;\mathrm{Day}1}$$

### Electron microscopy

After overnight culture, both U87 and U87_TRAF3IP2KD_ cells were fixed in 1% glutaraldehyde for 16 h at 4° C. The cryogenic system Gatan Alto 2500 and the Hitachi S-4800 Field Emission Scanning Electron Microscope were used for cryo scanning electron microscopy and imaging.

### Cell cycle analysis

U87_TRAF3IP2KD_ and U87_control shRNA_ cells were mixed with a lysing and permeabilizing agent (DNA PREP Reagent Kit; Beckman Coulter) and then stained and analyzed using a Cytomics FC 500 flow cytometer (Beckman Coulter). The cell cycle distribution was calculated using ModFit LT software version 3.2 (Verity Software House). The experiment was performed in triplicate.

### Microarray hybridization and data analysis

Microarray-based analysis of transcriptome profiles in U87_TRAF3IP2KD_ and U87_control shRNA_ cells was performed using Human Gene 2.0 ST array. Total RNA was isolated from U87_TRAF3IP2KD_ and U87_control shRNA_ cells and was used to generate biotin-labeled cRNA (BioArray HighYield RNA Transcription Labeling kit; Enzo Diagnostics). The biotinylated cRNA was cleaned (RNAeasy Mini kit; Qiagen), fragmented, and hybridized on GeneChips (triplicates/cell type). Human Gene 2.0 ST CEL files were normalized to produce gene-level expression values using the implementation of Robust Multiarray Average (RMA) in the affy package (version 1.36.1) [45] included in the Bioconductor software suite (version 2.12) [46] and an Entrez Gene-specific probe set mapping (17.0.0) from the Brainarray, University of Michigan [47]. Array quality was assessed by computing Relative Log Expression (RLE) and Normalized Unscaled Standard Error (NUSE) using the affyPLM package (version 1.34.0) [48]. Principal Component Analysis (PCA) was performed using the prcomp R function with expression values that had been normalized across all samples to a mean of zero and a standard deviation of one. Differential expression was assessed using the moderated (empirical Bayesian) *t* test implemented in the limma (Linear Models for Microarray and RNA-Seq) data package (version 3.14.4) (*i.e*., creating simple linear models with lmFit, followed by empirical Bayesian adjustment with eBayes). Analyses of variance were performed using the f.pvalue function in the sva package (version 3.4.0). Correction for multiple hypothesis testing was accomplished using the Benjamini-Hochberg false discovery rate (FDR) [49]. To perform these comparisons, probe sets whose target was not detected in any sample were eliminated from the data matrix. These criteria ensured that only those genes whose expression were not only highly differential between experiments but which were also expressed in a statistically significant manner. Genes that exhibited highly inconsistent expression patterns, as well as genes that did not exhibit any change in expression between different cell types, were excluded from the data matrix.

### Total RNA isolation and RT-qPCR

Total RNA extracted using the RNeasy Mini Extraction Kit (Qiagen) was converted to cDNA and used in qPCR in triplicates. GAPDH served as an invariant control. Fold changes were calculated using the ΔΔCT method. Human TRAF3IP2 and human GAPDH primers were obtained from Qiagen, and are considered proprietary. All other primers are listed in Table 1.

**Table 1:** List of primer sequences

VEGFA	5′GCGCTGATAGACATCCATGA-3′ and 5′-CCATGAACTTTCTGCTGTCTTG-3′
IL-1β	5′-GAACAAGTCATCCTCATTGCC-3′ and 5′-CAGCCAATCTTCATTGCTCAAG-3′
IL-6	5′-GCAGATGAGTACAAAAGTCCTGA-3′ and 5′-TTCTGTGCCTGCAGCTTC-3′
IL-8	5′-CTTCACACAGAGCTGCAGAA-3′ and 5′-GAGACAGCAGAGCACACAAG-3′
IL-10	5′-TCACTCATGGCTTTGTAGATGC-3′ and 5′-GCGCTGTCATCGATTTCTTC-3′
NF-κB	5′-GCAAAGGGAACATTCCGATAT-3′ and 5′-GCGACATCACATGGAAATCTA-3′
CCND1	5′-AGCGGTCCAGGTAGTTCA-3′ and 5′-GTGTCCTACTTCAAATGTGTGC-3′
IL-17 R	5′-CATGAACTCTGTCCCCATCC-3′ and 5′-CCCACGGACACCAGTATCTT-3′

### Chemiluminescent cytokine array

After seeding U87 and U87_TRAF3IP2KD_ cells (4 × 10^5^ cells) for 24 hours, the complete media was replaced with medium containing no serum and incubated for 16 h. The culture medium was collected, spun at 1000 × g for 10 minutes, and then used for the array (Human Cytokine Array Panel A, R&D Systems) that includes C5/C5a, CD40 Ligand, G-CSF, GM-CSF, GROα, I-309, sICAM-1, IFN-γ, IL-1α, IL-1β, IL-1ra, IL-2, IL-4, IL-5, IL-6, IL-8, IL-10, IL-12 p70, IL-13, IL-16, IL-17, IL-17E, IL-23, IL-27, IL-32α, IP-10, I-TAC, MCP-1, MIF, MIP-1α, MIP-1β, Serpin E1, RANTES, SDF-1, TNF-α and sTREM-1. The membranes were imaged and analyzed by NIH Image J software. After the integrated density of every spot was calculated, the average of the duplicate spots was used to determine the cytokine level.

### ELISA of phosphorylated p65 protein levels

Phospho-p65 levels were quantified using the PathScan^®^ Phospho-NF-κB p65 (p-p65, Ser536) Sandwich ELISA Kit (Cell Signaling Technology, Inc.). Wild type U87 and U87_TRAF3IP2KD_ cells (1 × 10^6^ cells) seeded for 24 hours were treated with either TNFa (10 ng/ml) or TNFa + IL-17 (10 ng/ml) for 12 hours then subjected to ELISA according to the manufacturer’s instructions. The absorbance was read at 450 nm and 650 nm (*n* = 3).

### RT^2^ PCR array

Gene expression was analyzed using the Human Tumor Metastasis RT^2^ Profiler PCR array (PAHS-028Z, Qiagen). The data were analyzed using the software provided by the manufacturer. A 2-fold cut-off was applied for significance.

### *In vivo* xenograft model

In order to demonstrate the causal role of TRAF3IP2 in glioblastoma, we used two different strategies. In both strategies, homozygous immunodeficient NIH-III mice were used. All animal protocols were approved by the Animal Care and Use Committee at the Tulane University School of Medicine in New Orleans, LA, USA, and conformed to the *Guide for the Care and Use of Laboratory Animals*, published by the National Institutes of Health (DRR/National Institutes of Health, 1996).

Strategy I-Proof-of-principle strategy: The mice were injected subcutaneously into the flank region with U87_TRAF3IP2KD_ (1 × 10^6^ cells in 50 μl PBS+50 μl Matrigel). As a control, U87_control shRNA_ cells were injected in a similar fashion. PBS served as a second control. Animals were sacrificed after 5 weeks, and tumor volume (*V*) calculated using calipers using the following equation: *V* = (a × b^2^)/2, where ‘a’ is the longer diameter and ‘b’ is the shorter diameter.

Strategy II-Translationally-relevant strategy: Tumors were induced by injecting wild type U87 cells transduced with a lentiviral vector expressing luciferase gene (1 × 10^6^ in 50 µl PBS+50 μl Matrigel) into the flank region as described in Strategy I. After 14 days, tumors were identified by imaging for luciferase. The animals were then divided into two groups; the experimental group was injected subcutaneously onto the tumor surface with TRAF3IP2 shRNA-LV (2 × 10^7^ viral particles). The control group received a similar amount of Scrambled shRNA-LV. Luciferase expression was analyzed weekly using the IVIS Lumina XRMS *In Vivo* Imaging System (PerkinElmer). Tumor size was measured twice a week with a caliper.

### Histology-human brain GBM tissue array

Brain glioblastoma tissue array that includes 11 cases of glioblastoma tissue cores (Biomax, BS17016c) was subjected to immunohistochemistry (IHC) for TRAF3IP2 localization or hematoxylin and eosin (H&E) staining using standard protocols.

### Histology-xenograft tumor tissues

The tumor tissues were dissected, fixed in 10% neutral buffered-formalin, embedded in paraffin, sectioned at 5 μm-thickness, and used for IHC or H&E according to standard protocols. For IHC, the tissue sections were analyzed using primary antibodies against TRAF3IP2 (Santa Cruz Biotechnology), IL-8, Ki67, or caspase 3 (Abcam). The sections were imaged using a Zeiss Axio scope microscope.

### Statistical analysis

The data were analyzed using Prism Graph Pad 6 software (GraphPad Software, Inc.). Two-sided, unpaired *t*-test and one-way ANOVA were used to analyze the data for significance. Kaplan-Meier survival plots were created. All experiments were performed in triplicate. *P* < 0.05 was considered significant. All microarray analyses were performed using R environment for statistical computing (version 2.15.1).

## SUPPLEMENTARY MATERIALS TABLES









## Abbreviations

Glioblastoma: Glioblastoma multiforme
TME: Tumor Microenvironment
TRAF3IP2: TRAF3 Interacting Protein 2
NF-κB: Nuclear Factor-kappa B
AP-1: Activator Protein 1
CSCs: Cancer stem cells
CDK: Cyclin-Dependent Kinase
