# Supplementary material for: TRAF3IP2, a novel therapeutic target in glioblastoma multiforme
Source: Oncotarget. 2018 Jul 3;9(51):29772–88. doi: 10.18632/oncotarget.25710 (PMC6049871; doi:10.18632/oncotarget.25710)
Supplement: Supplementary file 1 [file oncotarget-09-29772-s001.pdf]

TRAF3IP2, a novel therapeutic target in glioblastoma multiforme

SUPPLEMENTARY MATERIALS

Supplementary Table 1: Fold change regulation of genes involve in cell cycle regulation (±2 fold change; *P* < 0.05).
See Supplementary_Table_1

Supplementary Table 2: Fold change regulation of genes involve in apoptosis pathway (±2 fold change; *P* < 0.05).
See Supplementary_Table_2

Supplementary Table 3: Fold change regulation of genes involve in extracellular matrix expression (±2 fold change; *P* < 0.05)

Gene Symbol	Fold change	*P* value
RPTN	9.0	1.2E-05
TIMP3	7.4	6.1E-08
COL1A2	6.3	1.1E-05
THBS1	5.2	1.1E-08
APOH	5.0	3.4E-06
HTRA1	5.0	5.2E-08
NDP	4.9	1.3E-06
MFAP4	4.7	2.9E-07
EFEMP1	4.6	3.9E-05
ADAMTSL1	4.5	1.5E-06
COL5A2	3.7	7.3E-05
MGP	3.6	1.4E-05
TNFRSF11B	3.4	2.4E-06
COL5A1	3.3	4.4E-06
FBN1	3.3	3.9E-06
MATN2	3.1	2.8E-07
RECK	3.0	3.5E-06
FN1	2.8	1.3E-06
GPC6	2.8	1.5E-05
WNT5B	2.7	4.6E-06
SPARC	2.7	3.9E-06
HMCN1	2.7	2.6E-05
NID2	2.6	2.4E-04
ADAMTS12	2.6	1.0E-06
IGFBP7	2.5	8.7E-07
COL6A1	2.4	1.0E-06
LAMA4	2.3	1.3E-03
SOD3	2.3	2.9E-04
LTBP2	2.3	6.7E-06
AGRN	2.3	1.8E-05
SPOCK1	2.3	7.5E-06
ECM2	2.2	4.5E-05
LAMC1	2.2	2.5E-05
COL8A1	2.2	4.5E-05
LEPRE1	2.2	1.3E-04
TGFB3	2.0	3.3E-05
TIMP2	2.0	2.1E-05
LOXL1	2.0	1.2E-03
ATP7A	2.0	1.6E-04
COL6A3	2.0	1.1E-03
MMP12	-2.2	3.2E-05
SERPINA1	-2.4	5.0E-05
ADAMTS5	-2.4	6.0E-05
MMP1	-3.7	7.3E-07
SHH	-4.2	1.5E-05

Supplementary Table 4: Fold change regulation of genes involve in metabolism pathway (±2 fold change; *P* < 0.05).
See Supplementary_Table_4
