# Supplementary material for: TRAF3IP2, a novel therapeutic target in glioblastoma multiforme
Source: Oncotarget. 2018 Jul 3;9(51):29772–88. doi: 10.18632/oncotarget.25710 (PMC6049871; doi:10.18632/oncotarget.25710)
Supplement: Supplementary file 2 [file oncotarget-09-29772-s002.docx]

**Supplementary Table 1**. Fold change regulation of genes involve in cell cycle regulation (±2 fold change; *P*<0.05)

| **Gene Symbol** | **Fold change** | ***P* value** |
| --- | --- | --- |
| IL12A | 7.4 | 1.9E-07 |
| THBS1 | 5.2 | 1.1E-08 |
| TUBA1A | 3.4 | 1.6E-03 |
| DBC1 | 3.4 | 3.3E-06 |
| GMNC | 2.8 | 2.6E-05 |
| RASSF4 | 2.5 | 8.9E-04 |
| DHCR24 | 2.3 | 5.6E-05 |
| SMAD6 | 2.3 | 5.1E-05 |
| SESN1 | 2.3 | 1.8E-05 |
| TUBG2 | 2.3 | 2.0E-05 |
| E2F7 | 2.2 | 1.0E-04 |
| RALB | 2.2 | 1.4E-05 |
| MUC1 | 2.1 | 1.4E-05 |
| PTPN3 | 2.1 | 2.3E-04 |
| MAPRE3 | 2.1 | 5.0E-05 |
| PRKAG2 | 2.1 | 1.0E-05 |
| CEP78 | -2.0 | 5.4E-05 |
| TICRR | -2.0 | 7.1E-05 |
| PARD6B | -2.0 | 5.5E-05 |
| CCP110 | -2.1 | 2.7E-05 |
| TP53INP1 | -2.1 | 7.7E-06 |
| CENPJ | -2.1 | 5.5E-05 |
| CCNF | -2.1 | 1.2E-04 |
| PIM1 | -2.1 | 2.5E-05 |
| E2F2 | -2.1 | 1.4E-05 |
| ASNS | -2.1 | 6.4E-06 |
| CDK6 | -2.1 | 2.3E-05 |
| BEX2 | -2.1 | 7.4E-04 |
| POLA1 | -2.1 | 2.0E-05 |
| MTBP | -2.2 | 7.8E-04 |
| DBF4B | -2.2 | 4.4E-05 |
| RCC1 | -2.2 | 2.2E-04 |
| RHNO1 | -2.2 | 1.0E-04 |
| INCENP | -2.2 | 6.9E-06 |
| GMNN | -2.3 | 1.6E-05 |
| HAUS2 | -2.3 | 3.7E-06 |
| NASP | -2.3 | 1.3E-04 |
| PPP1R15A | -2.3 | 3.9E-05 |
| FEN1 | -2.3 | 8.1E-06 |
| PCNA | -2.3 | 1.3E-06 |
| SESN2 | -2.3 | 2.2E-06 |
| RFC5 | -2.4 | 2.9E-06 |
| BTG3 | -2.4 | 1.7E-06 |
| RFC2 | -2.4 | 2.7E-06 |
| CDK2 | -2.4 | 6.8E-05 |
| RFWD3 | -2.4 | 4.5E-06 |
| KIF18A | -2.4 | 3.3E-06 |
| TSPYL2 | -2.4 | 6.3E-06 |
| CIT | -2.4 | 2.4E-06 |
| MASTL | -2.5 | 3.6E-06 |
| CEP57 | -2.5 | 6.2E-06 |
| ECT2 | -2.5 | 2.2E-06 |
| PID1 | -2.5 | 7.8E-06 |
| KNTC1 | -2.5 | 5.4E-06 |
| RBL1 | -2.5 | 2.6E-05 |
| NGF | -2.5 | 2.4E-05 |
| CDC25A | -2.5 | 1.6E-05 |
| CENPO | -2.5 | 7.0E-06 |
| MCM8 | -2.6 | 3.6E-06 |
| GINS2 | -2.6 | 1.4E-06 |
| RFC4 | -2.6 | 7.7E-06 |
| CHAF1A | -2.6 | 2.3E-06 |
| CDC14A | -2.6 | 3.4E-06 |
| CYLD | -2.6 | 2.5E-06 |
| ORC6 | -2.7 | 2.3E-06 |
| SKA2 | -2.7 | 1.3E-05 |
| GINS1 | -2.7 | 2.4E-05 |
| POLE | -2.7 | 2.7E-06 |
| UHRF1 | -2.7 | 2.0E-06 |
| IRF1 | -2.8 | 4.7E-05 |
| MCM4 | -2.8 | 5.9E-05 |
| KIF18B | -2.8 | 4.3E-06 |
| CDT1 | -2.8 | 9.0E-06 |
| CHAF1B | -2.8 | 5.1E-05 |
| ING1 | -2.9 | 2.5E-06 |
| FGF2 | -2.9 | 9.1E-07 |
| RACGAP1 | -2.9 | 1.3E-06 |
| PHGDH | -3.0 | 1.5E-06 |
| BARD1 | -3.0 | 7.9E-07 |
| CENPH | -3.0 | 4.8E-05 |
| CUL1 | -3.0 | 3.8E-07 |
| SGOL2 | -3.1 | 5.6E-06 |
| PRIM1 | -3.2 | 4.1E-05 |
| ERCC6L | -3.2 | 5.4E-05 |
| CENPM | -3.3 | 1.4E-04 |
| ORC1 | -3.3 | 1.6E-05 |
| MCM2 | -3.3 | 1.8E-06 |
| BRCA2 | -3.3 | 7.1E-06 |
| CENPN | -3.3 | 4.3E-06 |
| C11orf82 | -3.4 | 1.3E-04 |
| GADD45B | -3.4 | 6.2E-06 |
| MCM3 | -3.4 | 9.3E-06 |
| ZWINT | -3.5 | 2.2E-06 |
| MAPK6 | -3.5 | 1.3E-07 |
| DDIT3 | -3.5 | 2.4E-06 |
| TACC3 | -3.6 | 5.7E-06 |
| SPC24 | -3.6 | 1.2E-05 |
| E2F1 | -3.7 | 2.7E-06 |
| BRCA1 | -3.7 | 1.2E-06 |
| CKS2 | -3.8 | 4.0E-06 |
| BUB1B | -3.8 | 3.2E-07 |
| FBXO5 | -3.9 | 4.9E-06 |
| CENPI | -3.9 | 2.3E-06 |
| CDCA5 | -4.0 | 4.4E-06 |
| CLSPN | -4.0 | 6.6E-06 |
| GINS4 | -4.1 | 8.9E-07 |
| CKS1B | -4.2 | 1.6E-05 |
| CENPW | -4.2 | 3.2E-07 |
| RFC3 | -4.3 | 1.5E-06 |
| CENPA | -4.3 | 7.4E-08 |
| BLM | -4.3 | 5.6E-08 |
| MCM5 | -4.3 | 9.0E-07 |
| SIRT1 | -4.3 | 6.0E-08 |
| CDCA8 | -4.4 | 5.5E-06 |
| KIF20B | -4.4 | 1.8E-05 |
| AURKA | -4.4 | 1.5E-05 |
| CDC25C | -4.5 | 9.9E-08 |
| NEK7 | -4.5 | 1.1E-06 |
| MELK | -4.6 | 2.7E-07 |
| MCM7 | -4.7 | 3.6E-06 |
| AURKB | -4.7 | 1.2E-06 |
| GTSE1 | -4.9 | 4.1E-06 |
| PLK4 | -4.9 | 1.2E-05 |
| CENPK | -4.9 | 4.0E-06 |
| KIF20A | -4.9 | 2.7E-05 |
| SPAG5 | -5.0 | 1.1E-07 |
| MAD2L1 | -5.0 | 1.3E-04 |
| DTL | -5.1 | 6.5E-05 |
| BIRC5 | -5.1 | 4.9E-06 |
| SKA1 | -5.2 | 5.1E-06 |
| UBE2C | -5.2 | 3.4E-08 |
| CDC6 | -5.3 | 7.8E-06 |
| NUF2 | -5.3 | 4.0E-06 |
| CYP1A1 | -5.3 | 4.5E-06 |
| CENPE | -5.3 | 2.2E-06 |
| HJURP | -5.3 | 6.7E-06 |
| CCNB2 | -5.3 | 6.7E-06 |
| CDC45 | -5.3 | 1.4E-07 |
| IQGAP3 | -5.4 | 4.1E-07 |
| CCNB1 | -5.5 | 4.9E-06 |
| E2F8 | -5.5 | 4.7E-07 |
| CENPF | -5.5 | 5.4E-06 |
| TTK | -5.7 | 2.4E-07 |
| CASC5 | -5.9 | 1.4E-05 |
| TYMS | -5.9 | 9.3E-07 |
| KIF23 | -6.2 | 4.2E-06 |
| PLK1 | -6.3 | 2.5E-05 |
| CCNA2 | -6.4 | 6.6E-06 |
| GAS2L3 | -6.5 | 8.4E-08 |
| MYBL2 | -6.5 | 2.4E-06 |
| NEK2 | -6.8 | 1.2E-06 |
| FANCI | -6.8 | 3.7E-07 |
| MCM10 | -6.9 | 3.9E-06 |
| MLF1IP | -7.0 | 1.4E-07 |
| CDKN3 | -7.1 | 1.3E-06 |
| DLGAP5 | -7.2 | 3.0E-05 |
| IL8 | -7.3 | 3.5E-05 |
| CCNE2 | -7.4 | 1.8E-05 |
| BUB1 | -7.4 | 1.3E-05 |
| TOP2A | -7.5 | 5.4E-06 |
| FOXM1 | -7.7 | 1.4E-06 |
| NDC80 | -7.8 | 6.5E-06 |
| ESCO2 | -7.9 | 3.7E-05 |
| CDC20 | -7.9 | 1.2E-05 |
| SGOL1 | -8.3 | 1.7E-07 |
| RRM2 | -8.9 | 6.0E-06 |
| SPC25 | -9.0 | 5.5E-07 |
| CDK1 | -9.3 | 8.3E-06 |
| KIF2C | -9.4 | 1.3E-06 |
| PTGS2 | -20.4 | 1.5E-07 |
