# Supplementary material for: TRAF3IP2, a novel therapeutic target in glioblastoma multiforme
Source: Oncotarget. 2018 Jul 3;9(51):29772–88. doi: 10.18632/oncotarget.25710 (PMC6049871; doi:10.18632/oncotarget.25710)
Supplement: Supplementary file 3 [file oncotarget-09-29772-s003.docx]

**Supplementary Table 2**. Fold change regulation of genes involve in apoptosis pathway (±2 fold change; *P*<0.05)

| **Gene Symbol** | **Fold change** | ***P* value** |
| --- | --- | --- |
| IL12A | 7.4 | 1.9E-07 |
| TGFBR2 | 6.0 | 8.9E-09 |
| HGF | 5.3 | 3.8E-06 |
| THBS1 | 5.2 | 1.1E-08 |
| APOH | 5.0 | 3.4E-06 |
| PRLR | 4.7 | 5.6E-08 |
| TGM2 | 4.6 | 1.2E-07 |
| DAPL1 | 4.3 | 3.1E-05 |
| DEPTOR | 3.6 | 3.8E-04 |
| TNFRSF11B | 3.4 | 2.4E-06 |
| CD74 | 3.4 | 1.8E-05 |
| GRIK2 | 3.3 | 1.3E-05 |
| CCL2 | 2.8 | 1.5E-06 |
| ELMO1 | 2.8 | 7.0E-05 |
| FAIM2 | 2.8 | 2.9E-06 |
| DUSP6 | 2.7 | 1.8E-06 |
| TP53I3 | 2.6 | 8.2E-05 |
| KIAA1324 | 2.6 | 8.7E-07 |
| SCN2A | 2.5 | 1.2E-04 |
| ECE1 | 2.4 | 5.0E-06 |
| ALDOC | 2.4 | 1.1E-04 |
| UNC13B | 2.4 | 6.8E-05 |
| DHCR24 | 2.3 | 5.6E-05 |
| SMAD6 | 2.3 | 5.1E-05 |
| CAT | 2.3 | 3.3E-06 |
| ADA | 2.3 | 2.2E-05 |
| MYO18A | 2.3 | 2.4E-05 |
| TIAM1 | 2.3 | 8.1E-06 |
| TGFA | 2.3 | 2.3E-04 |
| AGRN | 2.3 | 1.8E-05 |
| SEMA3A | 2.2 | 6.4E-06 |
| RALB | 2.2 | 1.4E-05 |
| GJA1 | 2.2 | 2.5E-05 |
| RFFL | 2.2 | 2.3E-05 |
| GRAMD4 | 2.1 | 4.9E-04 |
| MUC1 | 2.1 | 1.4E-05 |
| CLIP3 | 2.1 | 3.6E-06 |
| APH1B | 2.0 | 1.1E-05 |
| CCKBR | 2.0 | 2.2E-05 |
| F2R | 2.0 | 5.3E-06 |
| VAV3 | 2.0 | 3.8E-05 |
| TGFB3 | 2.0 | 3.3E-05 |
| CSF2 | -2.0 | 1.4E-05 |
| TRAF1 | -2.0 | 2.7E-04 |
| IER3 | -2.0 | 1.6E-04 |
| PREX1 | -2.1 | 1.2E-05 |
| CBX4 | -2.1 | 6.4E-06 |
| TP53INP1 | -2.1 | 7.7E-06 |
| TJP2 | -2.1 | 5.4E-06 |
| PIM1 | -2.1 | 2.5E-05 |
| E2F2 | -2.1 | 1.4E-05 |
| ASNS | -2.1 | 6.4E-06 |
| KDR | -2.1 | 6.3E-06 |
| BEX2 | -2.1 | 7.4E-04 |
| BCL11B | -2.2 | 4.6E-06 |
| ATG3 | -2.3 | 2.2E-06 |
| PPP1R15A | -2.3 | 3.9E-05 |
| NRG1 | -2.3 | 5.0E-06 |
| NFKB1 | -2.3 | 5.2E-06 |
| PEG10 | -2.3 | 1.3E-05 |
| TRAIP | -2.4 | 9.1E-06 |
| NFKBIA | -2.4 | 1.5E-05 |
| FAM188A | -2.4 | 1.8E-05 |
| ECT2 | -2.5 | 2.2E-06 |
| XRCC2 | -2.5 | 1.7E-04 |
| NGF | -2.5 | 2.4E-05 |
| ESPL1 | -2.5 | 2.1E-05 |
| CKAP2 | -2.6 | 2.0E-05 |
| IL1B | -2.6 | 2.4E-04 |
| CYLD | -2.6 | 2.5E-06 |
| BTG1 | -2.6 | 4.5E-05 |
| DDIT4 | -2.7 | 3.8E-07 |
| IRF1 | -2.8 | 4.7E-05 |
| ITGA1 | -2.8 | 9.4E-07 |
| DRAM1 | -2.9 | 1.3E-06 |
| FGF2 | -2.9 | 9.1E-07 |
| NET1 | -2.9 | 9.5E-07 |
| BARD1 | -3.0 | 7.9E-07 |
| EPHA7 | -3.0 | 4.0E-06 |
| CUL1 | -3.0 | 3.8E-07 |
| HMOX1 | -3.1 | 6.8E-07 |
| BRCA2 | -3.3 | 7.1E-06 |
| C11orf82 | -3.4 | 1.3E-04 |
| GADD45B | -3.4 | 6.2E-06 |
| TNFSF14 | -3.5 | 1.8E-06 |
| EDN1 | -3.5 | 3.1E-07 |
| ADM | -3.6 | 1.1E-06 |
| HSPA1B | -3.6 | 4.7E-07 |
| STIL | -3.6 | 9.7E-06 |
| E2F1 | -3.7 | 2.7E-06 |
| BRCA1 | -3.7 | 1.2E-06 |
| TNFRSF9 | -3.7 | 1.0E-06 |
| TNFAIP3 | -3.8 | 3.8E-07 |
| BUB1B | -3.8 | 3.2E-07 |
| HMGB2 | -4.0 | 4.5E-07 |
| ZC3H12A | -4.0 | 1.9E-07 |
| CLSPN | -4.0 | 6.6E-06 |
| HELLS | -4.1 | 2.7E-05 |
| RNF144B | -4.1 | 3.1E-07 |
| SHH | -4.2 | 1.5E-05 |
| LMNB1 | -4.3 | 6.3E-05 |
| SIRT1 | -4.3 | 6.0E-08 |
| SERPINB2 | -4.4 | 4.4E-04 |
| GCH1 | -4.4 | 1.6E-06 |
| SLIT2 | -4.5 | 2.9E-06 |
| MELK | -4.6 | 2.7E-07 |
| PLA2G4A | -4.6 | 1.1E-07 |
| AURKB | -4.7 | 1.2E-06 |
| CADM1 | -4.9 | 3.1E-04 |
| MAD2L1 | -5.0 | 1.3E-04 |
| TRIB3 | -5.1 | 1.2E-07 |
| BIRC5 | -5.1 | 4.9E-06 |
| BIRC3 | -5.3 | 3.9E-05 |
| GULP1 | -5.7 | 1.0E-07 |
| TPX2 | -6.0 | 2.1E-06 |
| PLK1 | -6.3 | 2.5E-05 |
| DHRS2 | -6.8 | 6.6E-07 |
| BUB1 | -7.4 | 1.3E-05 |
| TOP2A | -7.5 | 5.4E-06 |
| CDK1 | -9.3 | 8.3E-06 |
| IL6 | -9.4 | 3.0E-09 |
| VNN1 | -12.0 | 4.0E-09 |
| IL24 | -13.8 | 3.8E-08 |
| PTGS2 | -20.4 | 1.5E-07 |
