# Supplementary material for: TRAF3IP2, a novel therapeutic target in glioblastoma multiforme
Source: Oncotarget. 2018 Jul 3;9(51):29772–88. doi: 10.18632/oncotarget.25710 (PMC6049871; doi:10.18632/oncotarget.25710)
Supplement: Supplementary file 4 [file oncotarget-09-29772-s004.docx]

**Supplementary Table 4**. Fold change regulation of genes involve in metabolism pathway (±2 fold change; *P*<0.05)

| **Gene Symbol** | **Fold change** | ***P* value** |
| --- | --- | --- |
| CPA4 | 7.9 | 1.9E-08 |
| TIMP3 | 7.4 | 6.1E-08 |
| LEPREL1 | 7.0 | 2.6E-06 |
| TGFBR2 | 6.0 | 8.9E-09 |
| THBS1 | 5.2 | 1.1E-08 |
| APOH | 5.0 | 3.4E-06 |
| PRLR | 4.7 | 5.6E-08 |
| TGM2 | 4.6 | 1.2E-07 |
| ADAMTSL1 | 4.5 | 1.5E-06 |
| ST3GAL5 | 4.5 | 1.8E-05 |
| SNAP25 | 4.0 | 2.3E-07 |
| CA9 | 3.9 | 1.9E-06 |
| ADAM19 | 3.8 | 2.5E-05 |
| ARHGAP29 | 3.8 | 7.1E-07 |
| ATP2B4 | 3.6 | 2.8E-07 |
| ABCC3 | 3.6 | 1.2E-06 |
| DHCR7 | 3.6 | 3.4E-07 |
| RDH5 | 3.6 | 2.9E-04 |
| YOD1 | 3.5 | 1.9E-06 |
| SULT1C2 | 3.5 | 7.8E-07 |
| NEK10 | 3.4 | 2.9E-06 |
| TUBA1A | 3.4 | 1.6E-03 |
| CPE | 3.3 | 2.3E-05 |
| AGPAT9 | 3.2 | 3.7E-06 |
| ADCY8 | 3.1 | 1.6E-06 |
| STS | 3.1 | 2.9E-07 |
| ABCA2 | 3.1 | 9.1E-06 |
| MYLK | 3.1 | 3.4E-07 |
| PON2 | 3.0 | 1.8E-07 |
| RECK | 3.0 | 3.5E-06 |
| NPTXR | 2.9 | 6.7E-06 |
| FYCO1 | 2.9 | 5.6E-06 |
| SPATA20 | 2.9 | 3.4E-05 |
| AK5 | 2.9 | 1.3E-05 |
| ST8SIA5 | 2.8 | 5.7E-04 |
| GM2A | 2.8 | 6.8E-07 |
| GAA | 2.8 | 8.2E-07 |
| HMGCS1 | 2.8 | 9.8E-05 |
| FHIT | 2.7 | 1.0E-04 |
| DHRS9 | 2.7 | 5.3E-04 |
| CHRM3 | 2.7 | 1.7E-06 |
| LDLR | 2.7 | 7.4E-06 |
| GALNT10 | 2.7 | 9.6E-07 |
| DNASE1L1 | 2.6 | 1.4E-05 |
| SC5DL | 2.6 | 1.0E-04 |
| TP53I3 | 2.6 | 8.2E-05 |
| LPCAT1 | 2.6 | 3.0E-05 |
| OTUB2 | 2.6 | 6.4E-07 |
| PDIA5 | 2.6 | 4.1E-06 |
| ADAMTS12 | 2.6 | 1.0E-06 |
| FMO4 | 2.6 | 2.5E-04 |
| PGAP3 | 2.6 | 3.6E-05 |
| MVK | 2.5 | 8.8E-06 |
| CROT | 2.5 | 2.2E-06 |
| PDGFRB | 2.5 | 3.0E-06 |
| GSTT1 | 2.5 | 4.2E-05 |
| UGT1A8 | 2.5 | 1.3E-02 |
| MVD | 2.5 | 2.9E-05 |
| ERMP1 | 2.5 | 4.5E-06 |
| GNG2 | 2.5 | 1.4E-06 |
| ARSG | 2.5 | 4.6E-06 |
| RNASEL | 2.5 | 4.9E-05 |
| PLA2G15 | 2.5 | 7.0E-06 |
| CYBRD1 | 2.5 | 1.0E-06 |
| LIPA | 2.4 | 3.9E-05 |
| CPQ | 2.4 | 5.6E-06 |
| ECE1 | 2.4 | 5.0E-06 |
| ALDOC | 2.4 | 1.1E-04 |
| UNC13B | 2.4 | 6.8E-05 |
| UROS | 2.4 | 2.6E-06 |
| GSTM2 | 2.4 | 1.7E-03 |
| ZNFX1 | 2.4 | 7.8E-06 |
| DHCR24 | 2.3 | 5.6E-05 |
| CLYBL | 2.3 | 7.1E-03 |
| CAT | 2.3 | 3.3E-06 |
| ADA | 2.3 | 2.2E-05 |
| DDAH1 | 2.3 | 1.3E-04 |
| MYO18A | 2.3 | 2.4E-05 |
| CYP2J2 | 2.3 | 3.9E-03 |
| LDLRAP1 | 2.3 | 7.9E-06 |
| GIP | 2.3 | 4.8E-05 |
| APOL2 | 2.3 | 5.0E-05 |
| QPRT | 2.3 | 2.6E-06 |
| AGRN | 2.3 | 1.8E-05 |
| SPOCK1 | 2.3 | 7.5E-06 |
| CPT2 | 2.2 | 1.9E-05 |
| NRXN2 | 2.2 | 1.9E-05 |
| MAN1B1 | 2.2 | 3.0E-06 |
| ALAD | 2.2 | 1.6E-05 |
| FUCA1 | 2.2 | 8.2E-06 |
| ADAM23 | 2.2 | 9.9E-06 |
| THSD4 | 2.2 | 4.5E-06 |
| TCN2 | 2.2 | 3.9E-06 |
| GLS | 2.2 | 9.4E-05 |
| MSMO1 | 2.1 | 2.8E-06 |
| STARD5 | 2.1 | 3.0E-04 |
| IDUA | 2.1 | 4.8E-04 |
| ARSI | 2.1 | 3.2E-05 |
| SQLE | 2.1 | 1.8E-04 |
| MUC1 | 2.1 | 1.4E-05 |
| GALNT11 | 2.1 | 6.6E-06 |
| B4GALNT1 | 2.1 | 8.4E-04 |
| ENDOD1 | 2.1 | 6.5E-05 |
| ZC4H2 | 2.1 | 1.2E-05 |
| NUDT12 | 2.1 | 2.3E-05 |
| ADAM12 | 2.1 | 7.0E-06 |
| RDH11 | 2.1 | 1.1E-05 |
| ORMDL3 | 2.1 | 6.7E-06 |
| IGFBP4 | 2.1 | 1.4E-04 |
| BLVRA | 2.1 | 3.8E-05 |
| ATP13A2 | 2.1 | 3.3E-05 |
| PRKAG2 | 2.1 | 1.0E-05 |
| ATP1A2 | 2.0 | 1.6E-04 |
| LHPP | 2.0 | 3.2E-03 |
| PRPS1 | 2.0 | 4.6E-06 |
| PYGL | 2.0 | 4.2E-04 |
| VAV3 | 2.0 | 3.8E-05 |
| ST6GALNAC6 | 2.0 | 4.4E-05 |
| HHIPL2 | 2.0 | 1.4E-04 |
| TIMP2 | 2.0 | 2.1E-05 |
| ODC1 | -2.0 | 1.6E-05 |
| CA2 | -2.0 | 4.7E-02 |
| PTGES3 | -2.0 | 3.0E-05 |
| DGKE | -2.0 | 4.1E-05 |
| RPL37 | -2.0 | 4.7E-04 |
| NUDT1 | -2.0 | 5.4E-04 |
| IER3 | -2.0 | 1.6E-04 |
| PREX1 | -2.1 | 1.2E-05 |
| ACYP1 | -2.1 | 6.0E-06 |
| DNAJA1 | -2.1 | 9.8E-06 |
| XRCC6BP1 | -2.1 | 1.9E-05 |
| THAP10 | -2.1 | 4.3E-05 |
| RNASEH2A | -2.1 | 9.1E-06 |
| ASNS | -2.1 | 6.4E-06 |
| SLC22A4 | -2.1 | 4.1E-05 |
| PLA2G4C | -2.1 | 7.0E-05 |
| ARSE | -2.1 | 4.0E-06 |
| POLA1 | -2.1 | 2.0E-05 |
| BCL11B | -2.2 | 4.6E-06 |
| CEBPG | -2.2 | 1.2E-05 |
| ENOSF1 | -2.2 | 5.5E-06 |
| PPIP5K2 | -2.2 | 1.9E-05 |
| INCENP | -2.2 | 6.9E-06 |
| MMP12 | -2.2 | 3.2E-05 |
| EDNRB | -2.3 | 4.6E-04 |
| HNRNPD | -2.3 | 8.4E-06 |
| PAFAH1B2 | -2.3 | 1.5E-04 |
| SLCO4A1 | -2.3 | 3.7E-05 |
| NFKB1 | -2.3 | 5.2E-06 |
| TPRA1 | -2.4 | 1.1E-06 |
| SLC7A5 | -2.4 | 2.3E-06 |
| NFKBIA | -2.4 | 1.5E-05 |
| CDK2 | -2.4 | 6.8E-05 |
| KIF18A | -2.4 | 3.3E-06 |
| EGR1 | -2.4 | 6.7E-05 |
| CIT | -2.4 | 2.4E-06 |
| ALG10 | -2.4 | 2.2E-05 |
| PFKFB3 | -2.4 | 6.8E-06 |
| ADAMTS5 | -2.4 | 6.0E-05 |
| EIF1AX | -2.5 | 1.1E-03 |
| PID1 | -2.5 | 7.8E-06 |
| KNTC1 | -2.5 | 5.4E-06 |
| AMPD3 | -2.5 | 1.2E-05 |
| EXOSC8 | -2.5 | 4.5E-06 |
| CENPO | -2.5 | 7.0E-06 |
| ESPL1 | -2.5 | 2.1E-05 |
| IL1B | -2.6 | 2.4E-04 |
| SLC3A2 | -2.6 | 1.0E-06 |
| SKA2 | -2.7 | 1.3E-05 |
| SRD5A2 | -2.7 | 2.2E-04 |
| DDIT4 | -2.7 | 3.8E-07 |
| UHRF1 | -2.7 | 2.0E-06 |
| LBR | -2.8 | 2.1E-05 |
| GGH | -2.8 | 1.2E-05 |
| EME1 | -2.9 | 3.1E-05 |
| RACGAP1 | -2.9 | 1.3E-06 |
| PHGDH | -3.0 | 1.5E-06 |
| CENPH | -3.0 | 4.8E-05 |
| SGOL2 | -3.1 | 5.6E-06 |
| ELOVL7 | -3.1 | 6.0E-07 |
| HMOX1 | -3.1 | 6.8E-07 |
| PRIM1 | -3.2 | 4.1E-05 |
| ERCC6L | -3.2 | 5.4E-05 |
| CENPM | -3.3 | 1.4E-04 |
| MCM2 | -3.3 | 1.8E-06 |
| BRCA2 | -3.3 | 7.1E-06 |
| CENPN | -3.3 | 4.3E-06 |
| ZWINT | -3.5 | 2.2E-06 |
| SNRPE | -3.5 | 8.2E-04 |
| EDN1 | -3.5 | 3.1E-07 |
| ADM | -3.6 | 1.1E-06 |
| HSPA1B | -3.6 | 4.7E-07 |
| SPC24 | -3.6 | 1.2E-05 |
| ALG10B | -3.7 | 1.8E-02 |
| MMP1 | -3.7 | 7.3E-07 |
| VAPA | -3.7 | 4.7E-07 |
| BUB1B | -3.8 | 3.2E-07 |
| FBXO5 | -3.9 | 4.9E-06 |
| CENPI | -3.9 | 2.3E-06 |
| CDCA5 | -4.0 | 4.4E-06 |
| ZC3H12A | -4.0 | 1.9E-07 |
| TMPPE | -4.0 | 2.5E-06 |
| NUPL1 | -4.1 | 3.9E-07 |
| SHH | -4.2 | 1.5E-05 |
| CENPA | -4.3 | 7.4E-08 |
| PDE1A | -4.3 | 1.1E-05 |
| SIRT1 | -4.3 | 6.0E-08 |
| CDCA8 | -4.4 | 5.5E-06 |
| GCH1 | -4.4 | 1.6E-06 |
| NEK7 | -4.5 | 1.1E-06 |
| EXO1 | -4.5 | 5.7E-07 |
| PLA2G4A | -4.6 | 1.1E-07 |
| TK1 | -4.6 | 6.7E-06 |
| AURKB | -4.7 | 1.2E-06 |
| GEN1 | -4.8 | 1.3E-06 |
| CENPK | -4.9 | 4.0E-06 |
| MAD2L1 | -5.0 | 1.3E-04 |
| TRIB3 | -5.1 | 1.2E-07 |
| BIRC5 | -5.1 | 4.9E-06 |
| SKA1 | -5.2 | 5.1E-06 |
| HES1 | -5.3 | 1.7E-05 |
| NUF2 | -5.3 | 4.0E-06 |
| CYP1A1 | -5.3 | 4.5E-06 |
| CENPE | -5.3 | 2.2E-06 |
| BIRC3 | -5.3 | 3.9E-05 |
| CCNB1 | -5.5 | 4.9E-06 |
| CENPF | -5.5 | 5.4E-06 |
| CASC5 | -5.9 | 1.4E-05 |
| TYMS | -5.9 | 9.3E-07 |
| PLK1 | -6.3 | 2.5E-05 |
| NEK2 | -6.8 | 1.2E-06 |
| DHRS2 | -6.8 | 6.6E-07 |
| MCM10 | -6.9 | 3.9E-06 |
| MLF1IP | -7.0 | 1.4E-07 |
| DLGAP5 | -7.2 | 3.0E-05 |
| BUB1 | -7.4 | 1.3E-05 |
| FOXM1 | -7.7 | 1.4E-06 |
| NDC80 | -7.8 | 6.5E-06 |
| ESCO2 | -7.9 | 3.7E-05 |
| CDC20 | -7.9 | 1.2E-05 |
| MKI67 | -8.0 | 1.6E-05 |
| SGOL1 | -8.3 | 1.7E-07 |
| RRM2 | -8.9 | 6.0E-06 |
| SPC25 | -9.0 | 5.5E-07 |
| KIF2C | -9.4 | 1.3E-06 |
| VNN1 | -12.0 | 4.0E-09 |
| ANGPT2 | -14.4 | 1.8E-07 |
| PTGS2 | -20.4 | 1.5E-07 |
